# Composition of plant-based diets and the incidence and prognosis of inflammatory bowel disease: a multinational retrospective cohort study

**DOI:** 10.1016/j.lanepe.2025.101264

**Published:** 2025-03-14

**Authors:** Jie Chen, Yuhao Sun, Lintao Dan, Judith Wellens, Shuai Yuan, Hong Yang, Tammy Y.N. Tong, Amanda J. Cross, Nikos Papadimitriou, Antoine Meyer, Christina C. Dahm, Susanna C. Larsson, Alicja Wolk, Jonas F. Ludvigsson, Kostas Tsilidis, Edward Giovannucci, Jack Satsangi, Xiaoyan Wang, Evropi Theodoratou, Simon S.M. Chan, Xue Li, EPIC Investigatorsaa, Marie-Christine Boutron-Ruault, Marcela Guevara, Marc J. Gunter, Mazda Jenab, Rudolf Kaaks, Tim J. Key, María Dolores Chirlaque López, Giovanna Masala, Bas Oldenburg, Anja Olsen, Elio Riboli, Carlotta Sacerdote, Matthias Schulze, Gianluca Severi, Anne Tjønneland, Ruth C. Travis, Rosario Tumino, Roel Vermeulen, W.M. Monique Verschuren, Nick Wareham

**Affiliations:** a Department of Gastroenterology, Third Xiangya Hospital, Central South University, Changsha, China; b Department of Big Data in Health Science School of Public Health and the Second Affiliated Hospital, Zhejiang University School of Medicine, Hangzhou, Zhejiang, China; c Department of Gastroenterology, The Second Affiliated Hospital, Zhejiang University School of Medicine, Hangzhou, China; d Department of Gastroenterology, Ruijin Hospital, Shanghai Jiao Tong University School of Medicine, Shanghai, China; e Department of Gastroenterology and Hepatology, Leuven University Hostpital, Leuven, Belgium; f Translational Gastroenterology Unit, Experimental Medicine Division, Nuffield Department of Medicine, University of Oxford, John Radcliffe Hospital, Oxford, UK; g Unit of Cardiovascular and Nutritional Epidemiology, Institute of Environmental Medicine, Karolinska Institutet, Stockholm, Sweden; h Department of Gastroenterology, Peking Union Medical College Hospital, Peking Union Medical College & Chinese Academy of Medical Science, Beijing, China; i Cancer Epidemiology Unit, Nuffield Department of Population Health, University of Oxford, Richard Doll Building, Old Road Campus, Oxford, UK; j School of Public Health and Department of Surgery and Cancer, Imperial College London, London, UK; k Nutrition and Metabolism Branch, International Agency for Research on Cancer, Lyon, France; l INSERM, Centre for Research in Epidemiology and Population Health, Institut Gustave Roussy, Université Paris Saclay, Villejuif, France; m Department of Public Health, Aarhus University, Bartholins Allé 2, 8000, Aarhus, Denmark; n Department of Surgical Sciences, Uppsala University, Uppsala, Sweden; o Department of Medical Epidemiology and Biostatistics, Karolinska Institutet, Stockholm, Sweden; p Department of Pediatrics, Orebro University Hospital, Orebro, Sweden; q Department of Epidemiology and Biostatistics, School of Public Health, Imperial College London, London, UK; r Department of Hygiene and Epidemiology, University of Ioannina School of Medicine, Ioannina, Greece; s Department of Epidemiology, Harvard T.H. Chan School of Public Health, Boston, MA, USA; t Department of Nutrition, Harvard T.H. Chan School of Public Health, Boston, MA, USA; u Centre for Global Health, Usher Institute, University of Edinburgh, Edinburgh, UK; v Department of Gastroenterology, Norfolk and Norwich University Hospital NHS Trust, Norwich, UK; w Department of Medicine, Bob Champion Research and Education Building, Norwich Medical School, University of East Anglia, Norwich, UK; x Department of Gastroenterology, University Hospital of Bicêtre, Assistance Publique-Hôpitaux de Paris, Université Paris-Saclay, Le Kremlin Bicêtre, France

**Keywords:** Inflammatory bowel disease, Plant-based diet, Incidence, Prognosis, Genetic susceptibility, Mediation analysis

## Abstract

**Background:**

Many currently proposed diets for inflammatory bowel disease (IBD) focus on increasing plant-based foods, although a vegetarian diet can still contain products such as emulsifiers and refined grains that are believed to negatively impact IBD incidence and progression. To better inform dietary management in IBD, we investigated the association between plant-based diets and the incidence and complications of IBD.

**Methods:**

We leveraged data from the UK Biobank (UKB, 2009–2022) including 187,888 participants free of IBD at baseline and the European Prospective Investigation into Cancer and Nutrition (EPIC, 1991–2010) cohort including 341,539 individuals free of IBD across centres among Denmark, France, Germany, Greece, Italy, the Netherlands, Sweden and UK. Healthy and unhealthy diets were characterised using plant-based diet indexes (PDIs); in individual participants, these were based on the 24-h dietary recalls for UKB and food frequency questionnaires for EPIC. The primary outcome was the incidence of IBD; secondary outcomes evaluated endpoints of disease prognosis (IBD-related surgery, diabetes, cardiovascular diease, and all-cause mortality). Cox regression was applied to estimate hazard ratios (HRs).

**Findings:**

In the UKB (925 incident IBD, median follow-up 11.6 years, IQR 1.3 years), higher adherence to healthy PDI was associated with a lower IBD risk (HR 0.75, 95% CI 0.60–0.94), while higher alignment to an unhealthy PDI associated with an increased risk (HR 1.48, 95% CI 1.21–1.82) when comparing extreme quintiles of PDIs. Among individuals with established IBD, healthy PDI was inversely associated (HR 0.50, 95% CI 0.30–0.83) and unhealthy PDI was positively associated (HR 2.12, 95% CI 1.30–3.44) with need for IBD-related surgery. We did not observe significant associations between PDIs and risk of cardiovascular disease, diabetes mellitus or mortality. In the EPIC study (548 incident IBD, median follow-up 14.5 years, IQR 7.0 years), the HR of incident IBD for healthy PDI was 0.71 (95% CI 0.59–0.85) and for unhealthy PDI was 1.54 (95% CI 1.30–1.84).

**Interpretation:**

We provide evidence that the composition of a plant-based diet may be an important determinant of the risk of developing IBD, and of disease course after diagnosis. Further research is needed to explore the mechanistic pathways linking plant-based diets and IBD incidence and prognosis.

**Funding:**

10.13039/100014717National Natural Science Foundation of China, 10.13039/501100019540Natural Science Fund for Distinguished Young Scholars of Zhejiang Province, National Undergraduate Training Program for Innovation and Entrepreneurship, CRUK Career Development Fellowship, The “Co-PI” project, Natural Science Fund for Excellent Young Scholars of Hunan Province.

**Research in context**

**Evidence before this study**

Inflammatory bowel disease (IBD) is a chronic incurable disease bringing with huge socioeconomic burden. Current evidence suggests that Western and animal-based diets are positively correlated with IBD incidence and unfavorable disease outcomes. This raises the intriguing possibility that plant-based diets may offer a dietary approach to IBD management. To investigate the association between plant-based diets and IBD, we conducted a systematic search in PubMed for publications up from database inception to Jul 20, 2024. The search terms include (‘inflammatory bowel disease’ OR ‘Crohn's disease’ OR ‘ulcerative colitis’) AND (‘plant-based diet’ OR ‘plant diet’), with no language restriction. Previous studies showed that adherence to plant-rich diet pattern is associated with lower incidence and better prognosis of IBD, but these studies did not consider the harm of animal-based foods. Research examining the effect of plant-based diet on the development and disease course of IBD is lacking.

**Added value of this study**

Based on data from two large cohorts of nearly one million people from multiple European countries, we reveal a lower risk of incident IBD and related surgery when consuming a healthy plant-based diet, whereas the inverse was true when following an unhealthy plant-based diet. These associations may be greater when at high genetic risk for IBD and are partially mediated by anti-inflammatory properties of the diet.

**Implications of all the available evidence**

We show that adherence to a healthy plant-based dietary index might be a strategy to alter the natural history of IBD, especially in individuals with moderate or high genetic risk. At the same time, we show that not all plant-based diets are equal. Clinically this underscores the need for specialised dietetic counselling to ensure the overall quality of the diet in IBD management, while future research efforts should focus on determining the different aspects within plant-based foods that explain this dichotomy to ensure healthy food in a sustainable environment.

## Introduction

Inflammatory bowel disease (IBD), including Crohn's disease (CD) and ulcerative colitis (UC), are chronic gastrointestinal diseases characterized by intestinal inflammation, abdominal pain and diarrhoea,^1^ with an increasing global disease burden.^2^ Understanding environmental factors that influence disease risk is essential for developing effective public health strategies to prevent disease onset and potentially improve disease outcomes.^3^

Based on epidemiological and interventional data,^4^, ^5^, ^6^ diet is believed to play a pivotal role in the pathogenesis and course of IBD.^7^ Typical Western diets, characterized by high intakes of ultra-processed and animal-based foods, are positively associated with both incidence^8^ and poor disease course^9^^,^^10^ of IBD. However, it is important to note that certain therapeutic dietary interventions, such as the Crohn's Disease Exclusion Diet (CDED), incorporate significant amounts of animal-based products for nutritional support during flares. Meanwhile, Bolte et al. showed that plant-based food consumption is associated with higher synthesis and conversion of essential nutrients (e.g., short chain fatty acid) by the gut microbiota in patients with IBD, while animal-derived food intake showed the opposite association.^11^ This raises the critical question of whether plant-based diets should be advised to patients with IBD and individuals at risk.

Plant-based diets have a flexible definition and can include vegan, vegetarian, or predominantly plant-based omnivorous patterns.^12^ The development of plant-based diet indexes (PDIs)—including the PDI, healthy PDI and unhealthy PDI—has provided a standardized method to assess adherence to plant-based eating patterns.^13^ These indexes enable nuanced analyses of dietary patterns, offering valuable tools for cross-cultural studies and dietary comparisons.^12^ Previous work has shown that plant-based diets could contribute to the management of IBD^1^ by supressing inflammation^14^ and modulating the gut microbiota. However, large-scale cohort studies evaluating the association between PDIs and both IBD incidence and prognosis are limited; yet European regions report the highest consumption of animal-based foods alongside a significant IBD burden.^2^^,^^15^ Moreover, existing studies have largely overlooked the complex interactions between diet and genetic susceptibility, which could inform more precise dietary recommendations tailored to individuals' genetic profiles.^16^^,^^17^

To address these gaps, we conducted a retrospective cohort study from two prospective cohort studies encompassing three sub-populations to: (1) evaluate associations between PDIs and incident IBD among individuals without pre-existing disease; (2) validate and assess the generalizability of these associations across different regions; and (3) investigate the relationship between PDIs and clinical outcomes in individuals with established IBD. We also conducted analysis to quantify mediation effect of the inflammatory markers in the associations between PDIs and incident IBD among individuals without pre-existing disease. Additionally, we also explored the interaction between PDIs and genetic susceptibility in risk of IBD incidence.

## Methods

### Study design and participants

The current analysis leveraged data from UK Biobank and the European Prospective Investigation into Cancer and Nutrition (EPIC) studies. The UK Biobank is a large population-based cohort with more than 500,000 participants aged 37–73 years, recruited from 22 assessment centres between 2006 and 2010.^18^ Participants in UK Biobank have received a web-based 24-h dietary recall (WebQ) administered in five rounds between 2009 and 2012 and were followed by national electronic health-related records up to 2022. The EPIC-IBD is a sub-cohort of the main EPIC study including participants without an IBD diagnosis at enrolment. EPIC-IBD study included participants aged 20–80 years old, recruited between 1991 and 1998 from multiple European countries (Denmark, France, Germany, Greece, Italy, the Netherlands, Sweden and UK).^19^ At baseline, participants provided detailed demographic, lifestyle, and dietary data using validated food frequency questionnaires (FFQs). The EPIC-IBD study used data from registries, follow-up questionnaires, and hospital databases until 2004–2010, varying by centres.

**Fig. 1:**
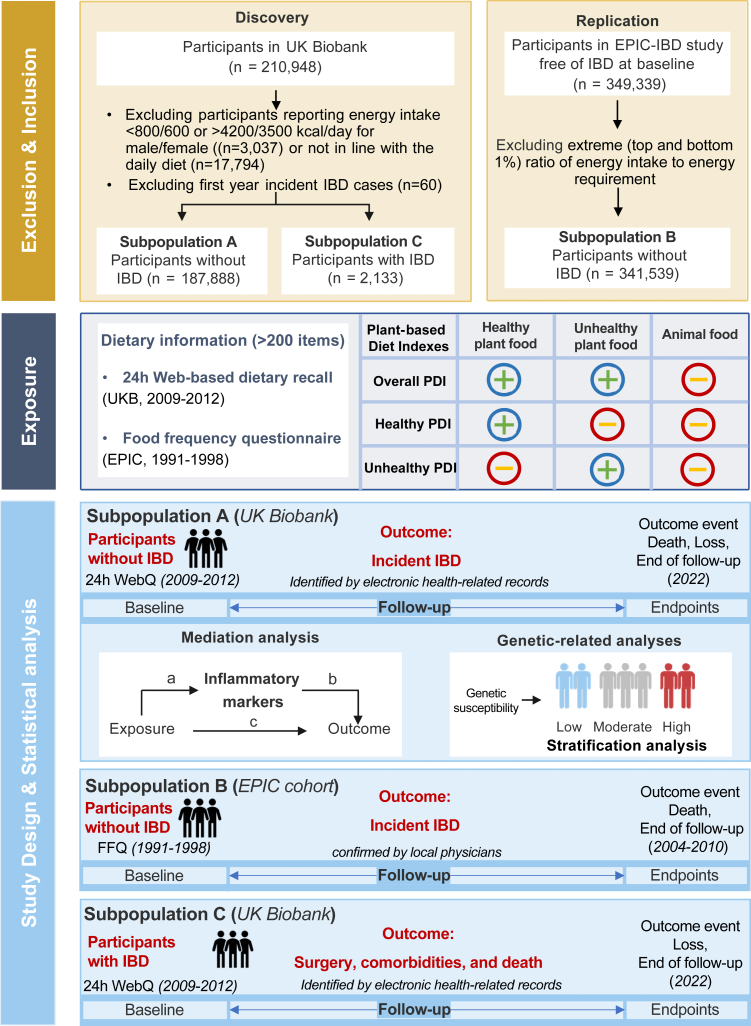
**Flowchart of the study in the UK Biobank (discovery cohort) and the European Prospective Investigation into Cancer and Nutrition study (replication cohort)**.

The study design is presented in Fig. 1. First, we investigated the association between PDIs and IBD among individuals free of IBD using UK Biobank in the first steps. Among 210,948 UK Biobank participants with available baseline 24-h dietary recalls, we excluded participants: (1) who reported that the questionnaire was not in line with their usual intake patterns (n = 17,794); (2) who reported an extreme energy intake (n = 3073, normal range: men with 800–4200 kcal/day, and women with 600–3500 kcal/day)^13^; (3) who were identified with IBD (n = 2133) diagnosed before baseline; (4) who were diagnosed with IBD within 1-year of follow-up (n = 60), leaving 187,888 participants for this analysis.

Second, we explored the external generalizability of PDIs and incident IBD associations across different European regions using the EPIC-IBD cohort (only including participants without an IBD diagnosis at enrolment). Among 349,339 available participants, we excluded those within an extreme (top and bottom 1%) ratio of energy intake to energy requirement (n = 7800),^20^ leaving 341,539 participants for analysis.

Finally, we included UK Biobank participants with valid dietary data and with IBD diagnosed before baseline (n = 2133) to investigate the associations between PDIs and prognosis endpoints of IBD. For each of the four prognosis outcomes, individuals with prevalent cases at baseline were excluded respectively. We did not replicate this step in EPIC-IBD cohort given the data availability.

The UK Biobank and EPIC have ethical approval from the North West Multi-Centre Research Ethics Committee and the local ethics committees, respectively and all involved subjects provided signed informed consent. More details of the EPIC and EPIC-IBD cohorts are described elsewhere.^21^^,^^22^ As all participants' data were anonymized and de-identified, no additional ethical applications were made for this study.

### Assessment of plant-based diet indexes (PDIs)

In the UK Biobank, dietary information was collected by a Web-based 24-h dietary recall (WebQ) administered in five rounds between 2009 and 2012, reporting daily intake of over 200 common foods and 30 beverages. The WebQ is in good agreement with long-term consumption and frequency of food groups collected by baseline FFQs.^23^ Compared to interviewer-administered 24-h recall completed on the same day, Spearman correlation coefficients calculated from the WebQ ranged from 0.5 to 0.9 (mean 0.6) for most nutrients.^24^ In the EPIC study, dietary information was assessed using validated country-specific food frequency questionnaires (FFQs) at baseline recruitment, evaluating the regular diet covering 98 to 260 food items during the preceding 12 months.^21^ Reported food items were categorized according to the harmonized food categories common to each questionnaire. In all recruitment centres, the FFQs were validated using 24-h recall questionnaires, with Spearman correlation coefficients ranging from 0.37 to 0.79 for food groups.^25^

**Fig. 2:**
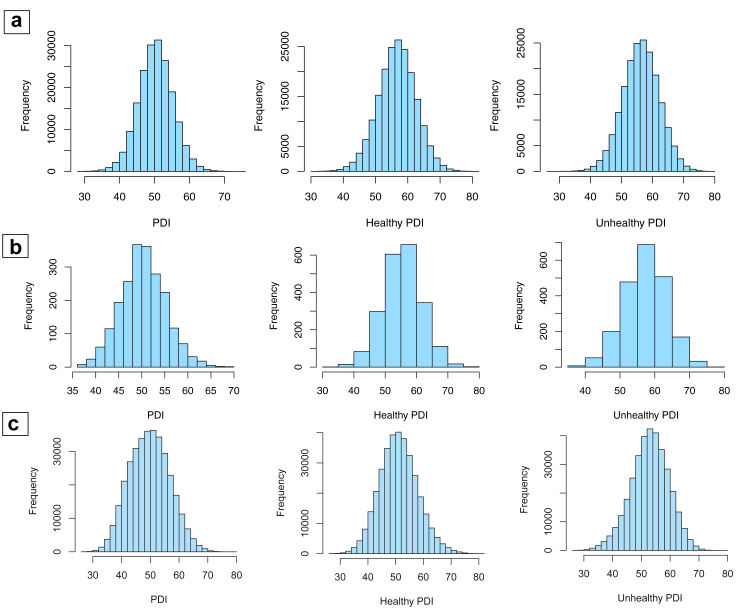
**Distributions of planted-based diet indexes among (a) participants free of IBD in the UK Biobank; (b) diagnosed as IBD at baseline in the UK Biobank; and (c) participants free of IBD in the European Prospective Investigation into Cancer and Nutrition study.** PDI, plant-based diet index; HR, hazard ratio; CI, confidence interval.

Three versions of the PDI (PDI, healthy PDI, and unhealthy PDI) were constructed by scoring the intake of 3 broad food groups (healthy plant foods, unhealthy plant foods, and animal foods) comprising 18 food groups.^26^^,^^27^ The overall PDI assigned positive scores to all plant foods. The healthy PDI assigned positive scores to healthy plant foods (whole grains, fruits, vegetables, nuts, legumes and vegetarian protein alternatives, as well as tea and coffee) and reverse scores to unhealthy plant foods (refined grains, potatoes/fries, fruit juices, sugar-sweetened beverages, sweets and desserts). The unhealthy PDI assigned positive scores to unhealthy plant foods and reverse scores to healthy plant foods. In all three PDIs, animal foods (animal fat, dairy, egg, fish or seafood, meat, and miscellaneous animal-based foods) were given reverse scores. For positive scores, the highest intake quintile scored 5 points and the lowest intake quintile scored 1 point, and vice versa. The distribution of PDI, healthy PDI, and unhealthy PDI scores is presented in Fig. 2. These indicators could present incremental dietary changes instead of defining the plant-based diet as vegetarian diets and dichotomizing study populations by consumption of animal foods. In addition, we also created a ‘healthy omnivorous diet’ based on the healthy PDI by assigning certain typically assumed healthy animal food (dairy, eggs, and fish or seafood) positive scores for sensitivity analysis.^13^ The common food items used to calculate the two cohorts were presented in the Supplementary method.

### Ascertainment of outcome

The primary outcome was the incidence of IBD. In the UK Biobank, the IBD events were ascertained by external linkage to national hospital inpatient records, primary care data, and the death registry. We extracted IBD diagnosis with the specific diagnostic code (International Classification of Disease, 10th [ICD-10] code: K50, K51; ICD-9 code: 555, 556). In the EPIC study, participants who developed incident IBD were identified either by self-administered follow-up questionnaires with medical records review by 1–2 physicians or national/regional registries, depending on the centre.^19^

The secondary outcomes were prognosis endpoints including comorbidities (cardiovascular diseases, diabetes mellitus), IBD-related outcomes (related surgery), and all-cause mortality. The surgery risk (including bowel resection and surgery for perineal disease) and all-cause mortality were recommended as important midterm and long-term complication measures by the SPIRIT expert consensus, respectively.^28^ Cardiovascular diseases and diabetes mellitus were considered emerging comorbidities in IBD^29^ and have been previously inversely linked to a plant-based dietary pattern in the general population.^27^^,^^30^ The definition of diagnosis details of each outcome is shown in Table S1. For each outcome, the individuals were followed from the baseline date (last completion date of dietary assessment) to the date of corresponding outcome incidence, loss to follow-up, death, or end of follow-up, whichever came first.

### Covariates

At recruitment, for both the UK Biobank and EPIC study data on physical examinations were collected as well as self-administered questionnaires. We included age (continuous), sex (female, male), Townsend deprivation index (TDI, a measure of material deprivation and positive values indicated high levels of poverty; For UK Biobank only), education (with or without university/college degree), ethnicity (white, non-white), body mass index (BMI, continuous), smoking status (never, ever, or current), alcohol consumption (ethanol, g/d), exercise time (minutes/week), total energy intake (KJ/d), total sugar intake (g/d), and the intake of ultra-processed food (based on the NOVA classifications,^31^ in servings/day) as covariates. BMI, smoking, physical activity, ultra-processed food, and sugar intake were established risk factors in the incidence and disease course of IBD by previous umbrella reviews.^32^^,^^33^ Regular use of IBD-related medication, use of aminosalicylates, corticosteroids, and immunomodulators, was obtained via verbal review.

Considering the potential roles of inflammation, we selected serum C-reactive protein (CRP) levels and INFLA-score (calculation details were presented in the Supplementary Method) as mediators as suggested by previous studies for our mediation analysis.^34^ Single imputation for missing values of all covariates (imputed to the median values for continuous variables or applied the most frequently used category for categorical variables) was applied given the low rate of the missing data (all <5%).

### Polygenic risk score

Previous studies have demonstrated the important role of genetic factors in the development of IBD^35^; therefore, we evaluated associations between PDIs and incident IBD among UK Biobank participants with different genetic susceptibility of IBD. Polygenic risk score (PRS) was applied to estimate the genetic susceptibility of IBD using independent single-nucleotide polymorphisms (SNP) that were identified as risk loci of IBD, CD, and UC by previous GWAS.^35^ The detailed method and genetic variants we used to calculate PRS are presented in the Supplementary Method. The derived PRS was divided into three groups (low, moderate, and high) by using tertiles. For genetic-related analysis in the UK Biobank, to ensure homogeneity, we further excluded participants of non-white ethnicity and without complete genetic information, leaving a total of 175,737 remaining participants.

### Statistical analysis

The primary analysis was conducted using data from the UK Biobank. Multivariable-adjusted Cox proportional hazard regression models were used to estimate hazard ratios (HR) and 95% confidence interval (CI) examining the association of PDI by quintiles or per 10-unit increment (approximately 2 SD) with the incidence and complications of IBD. The lowest categories of three PDI scores were considered as the corresponding reference categories, respectively. Two multivariable-adjusted models were constructed: model 1 (minimally adjusted) adjusted for age and sex; model 2 (fully adjusted) further adjusted for TDI, education, ethnicity, BMI, smoking status, alcohol consumption, exercise time, total energy intake, and total sugar intake. All models satisfied the proportional hazards assumption tested by the Schoenfeld residuals (*P* > 0.05). The trend across quintile groups of PDIs was estimated using the median of each group. To visualize the dose–response associations, restricted cubic splines with three knots placed at the 10th, 50th^,^ and 90th percentiles of PDIs were used. We conducted analyses to explore the external validity of the association between PDI and risk of incident IBD in the EPIC cohort. Multivariable Cox regression models with similar covariates adjusted in discovery analysis were used to evaluate the HR and 95% CIs. We also combined the results of the two cohorts using the R package “meta” using fixed model. Cochran's Q test was used to assess the difference in the estimates.

The associations between individual food groups and incident IBD were explored. We also investigated the associations of PDIs with incident CD and UC as well as associations of PDIs with prognosis endpoints in individuals with CD and UC.

Mediation analysis was conducted to quantify the contribution of inflammation in the significant associations of PDIs with the incidence or prognosis of IBD identified in the primary analysis. Mediation analysis distinguishes the direct effect of PDIs on the incidence or prognosis of IBD, and the indirect effect mediated by inflammation status. Two markers (CRP levels or INFLA-score) were chosen as mediators to represent inflammation status. This analysis used the ‘Mediation’ package in R.^36^ The proportion of associations mediated by selected inflammation-related mediators was calculated as [indirect effect/(indirect + direct effect)]. Bootstrap with 5000 simulations was used to calculate the corresponding confidence intervals and *P*-values. We restricted the analysis to individuals with available CRP levels or INFLA-score.

We conducted a stratification analysis to examine the associations between PDIs and incident IBD stratified by genetic risk groups (low, moderate, and high) defined as tertiles of the PRS scores. We further adjusted the first 5 principal components of ancestry based on the fully adjusted model (model2) when conducted this analysis. The multiplicative interaction was evaluated by testing the change of models before and after allowing a multiplication term of the PDIs and the genetic risk group based on the likelihood ratio test.

We conducted the following sensitivity analyses to test the robustness of the primary finding: 1) restrict the analysis to participants with at least two dietary recalls; 2) evaluate the effects of healthy omnivorous diet on the risk of incident IBD and evaluate this association with additional adjustment of dietary fibre intake; 3) additionally adjusted for ultra-processed food intake; 4) additionally adjusted for IBD-related medication for associations between PDIs and prognosis endpoints; 5) consider death as competing events when investigating the remaining outcomes of interest.

All analyses were performed using R software (version 4.2.1), and a two-sided *P* value < 0.05 was deemed significant.

### Role of the funding source

The funder had no role in study design, data collection and analysis, data interpretation, writing of the report, decision to publish, or preparation of the manuscript.

## Results

### PDI and risk of incident IBD

**Fig. 3:**
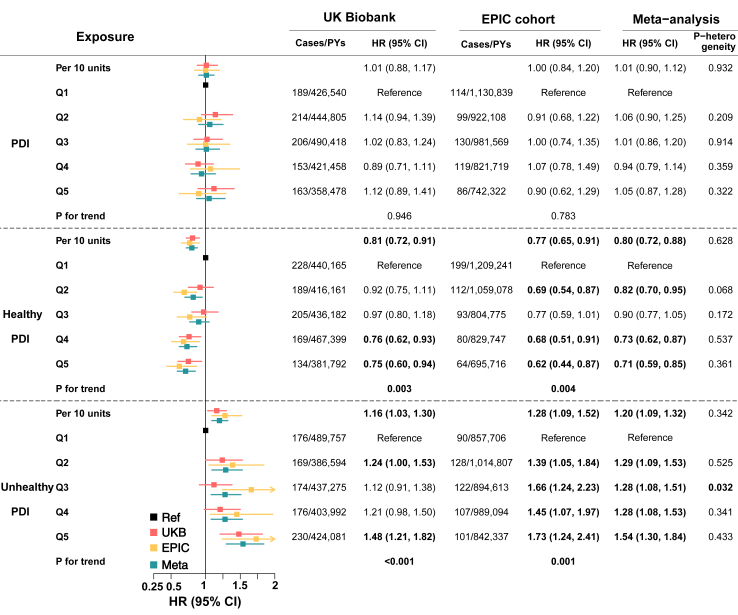
**Associations between the plant-based diet indexes and incident inflammatory bowel disease in UK Biobank (n = 187,888), EPIC cohort (n = 341,539), and the meta-analysis of the results from the two cohorts (n = 529,427).** HRs were adjusted for age, sex, Townsend deprivation index, education, ethnicity, body mass index, smoking status, alcohol consumption, exercise time, total energy intake and total sugar intake in UK Biobank. HRs were adjusted for age, sex, centre, education level, BMI, smoking status, alcohol consumption, total sugar, total energy, and physical activity measured in the EPIC study. The 20%, 40%, 60% and 80% percentile of PDIs in the UK Biobank and EPIC study were used as cut-off values as follow: PDI: (UKB) 46.8, 49.5, 52.0, 55.0; (EPIC) 44.0, 48.0, 52.0, 56.0; healthy PDI: (UKB) 52.0, 55.7, 58.5, 62.0; (EPIC) 46.0, 50.0, 53.0, 57.0; unhealthy PDI: (UKB) 52.0, 55.0, 58.0, 61.5; (EPIC) 48.0, 52.0, 55.0, 59.0. Numbers in bold indicates significant associations. P for heterogeneity were tested using Cochran Q test. PDI, plant-based diet index; HR, hazard ratio; CI, confidence interval; EPIC, European Prospective Investigation into Cancer, and Nutrition; PY, person-years.

Table 1 shows the baseline characteristics of participants in the UK Biobank by healthy PDI quintiles. Individuals with higher scores of PDI were more likely to be female, and have lower BMI, serum CRP levels, and INFLA-scores. Over 2,141,699 person-years of follow-up, 925 incident IBD (286 CD and 639 UC) cases were documented in UK Biobank. Comparing the extreme quintiles of PDIs, we observed an inverse association of the healthy PDI (HR: 0.75, 95% CI 0.60–0.94; *P* for trend = 0.003) and a positive association of the unhealthy PDI with IBD incidence (HR 1.48, 95% CI 1.21–1.82; *P* for trend = 0.001) (Fig. 3). These observed associations with PDIs were similar for CD and UC (Figures S1 and S2). We did not observe any associations between the overall PDI and IBD risk in either statistical model.

**Table 1:** Characteristics of participants by healthy plant-based diet index quintiles in the UK Biobank.^a^

Characteristics	Overall (n = 187,888)	Q1 (n = 38,868, ≤52.0)	Q2 (n = 36,570, >52.0–≤55.7)	Q3 (n = 38,252, >55.7–≤58.5)	Q4 (n = 40,854, >58.5–≤62.0)	Q5 (n = 33,344, >62.0)
**Age at baseline (mean (SD))**	50.70 (4.90)	48.81 (4.92)	49.72 (4.72)	50.34 (4.62)	51.32 (4.52)	53.62 (4.36)
**Sex (%)**^b^						
Female	103,326 (55.0)	15,887 (40.9)	18,247 (49.9)	21,502 (56.2)	25,035 (61.3)	22,655 (67.9)
Male	84,562 (45.0)	22,981 (59.1)	18,323 (50.1)	16,750 (43.8)	15,819 (38.7)	10,689 (32.1)
**Ethnicity (%)**^c^						
White	179,629 (95.6)	36,932 (95.0)	35,010 (95.7)	36,681 (95.9)	39,165 (95.9)	31,841 (95.5)
Others	8259 (4.4)	1936 (5.0)	1560 (4.3)	1571 (4.1)	1689 (4.1)	1503 (4.5)
**Townsend deprivation index (mean (SD))**	−1.60 (2.86)	−1.44 (2.95)	−1.62 (2.87)	−1.67 (2.82)	−1.67 (2.81)	−1.58 (2.86)
**Education (%)**						
Below college degree	107,058 (57.3)	24,857 (64.3)	21,437 (58.9)	21,624 (56.8)	22,256 (54.7)	16,884 (50.8)
College degree	79,924 (42.7)	13,792 (35.7)	14,931 (41.1)	16,442 (43.2)	18,430 (45.3)	16,329 (49.2)
**BMI, kg/m**^**2**^**, (mean (SD))**	26.91 (4.63)	27.94 (4.98)	27.16 (4.61)	26.83 (4.53)	26.49 (4.43)	26.03 (4.34)
**Smoking status (%)**						
Never	106,485 (56.8)	21,801 (56.2)	20,568 (56.4)	21,709 (56.9)	23,425 (57.5)	18,982 (57.1)
Previous	66,525 (35.5)	13,055 (33.7)	12,916 (35.4)	13,563 (35.6)	14,621 (35.9)	12,370 (37.2)
Current	14,396 (7.7)	3916 (10.1)	2980 (8.2)	2870 (7.5)	2718 (6.7)	1912 (5.7)
**Current drinkers (%)**	176,011 (93.7)	36,218 (93.2)	34,423 (94.1)	36,042 (94.2)	38,298 (93.7)	31,030 (93.1)
**Alcohol, g/d, (mean (SD))**	14.51 (19.96)	15.71 (22.45)	15.63 (20.58)	14.97 (19.84)	13.91 (18.69)	12.08 (17.43)
**Exercise time, minutes/day, (mean (SD))**	11.89 (8.84)	11.57 (9.54)	11.57 (8.77)	11.74 (8.54)	12.02 (8.55)	12.61 (8.71)
**CRP, mg/L, (mean (SD))**	2.31 (4.01)	2.65 (4.32)	2.42 (4.16)	2.30 (3.90)	2.17 (3.96)	1.95 (3.58)
**INFLA-score, (mean (SD))**	−0.70 (6.01)	0.14 (6.05)	−0.42 (6.00)	−0.70 (5.97)	−1.08 (5.97)	−1.51 (5.93)
**Total sugar, g/d, (mean (SD))**	119.3 (46.6)	119.1 (48.9)	116.0 (45.9)	117.0 (45.0)	119.7 (45.5)	125.9 (46.8)
**Total energy, KJ/d, (mean (SD))**	8587 (2298)	9617 (2427)	8792 (21,91)	8437 (2157)	8124 (2153)	7901 (2136)
**Healthy PDI score, (mean (SD))**	57.0 (5.8)	48.9 (3.0)	54.1 (0.9)	57.1 (0.8)	60.3 (1.1)	65.5 (2.7)

PDI, plant-based diet index; TDI, Townsend deprivation index; BMI, body mass index; CRP, C-reactive protein.

a Mean (SD) values and percentages are reported for continuous and categorical variables, respectively.

b The sex information was first ascertained by National Health System records and was verified by self-reported information of participants at baseline recruitment centers.

c Ethnicity was categorized into “White” (White, British, Irish, and any other White background) and “Others” (Mixed, Asian or Asian British, Black or Black British, Chinese, and other ethnic groups), based on the self-reported items. Ethnicity other than White was categorized as “Others” due to the small number of participants.

We included 341,539 individuals in the EPIC-IBD cohort and followed them for a median period of 14.5 years (4,598,557 person-years in total, IQR = 1.3 years). The characteristics are presented in Table 2. We documented 548 IBD (156 CD and 392 UC) cases during follow-up. The replicated associations of PDIs with incident IBD (Fig. 3) and its subtypes (Figures S1 and S2) in the EPIC cohort and the combined results of the two cohorts remained consistent with the primary analysis. Specially, the HRs of IBD for comparing extreme quintiles of healthy PDI (HR 0.71, 95% CI 0.59–0.85, *P* = 0.0002) and unhealthy PDI (HR 1.54, 95% CI 1.30–1.84, *P* < 0.0001) were comparable to results in UK Biobank.

**Table 2:** Characteristics of participants by healthy plant-based diet index quintiles in the EPIC cohort.^a^

Characteristics	Overall (n = 341,539)	Q1 (n = 77,986, ≤46.0)	Q2 (n = 74,073, >46.0–≤50.0)	Q3 (n = 59,440, >50.0–≤53.0)	Q4 (n = 64,962, >53.0–≤57.0)	Q5 (n = 65,078, >57.0)
**Age at baseline (mean (SD))**	52.1 (9.4)	51.7 (9.0)	52.6 (8.8)	52.8 (8.9)	52.8 (9.2)	50.6 (10.9)
**Sex (%)**^b^						
Female	238,874 (69.9)	42,506 (54.5)	49,890 (67.4)	43,330 (72.9)	50,216 (77.3)	52,932 (81.3)
Male	102,665 (30.1)	35,480 (45.5)	24,183 (32.6)	16,110 (27.1)	14,746 (22.7)	12,146 (18.7)
**Education (%)**						
None	12,769 (3.8)	4813 (6.3)	3236 (4.5)	2094 (3.6)	1774 (2.8)	852 (1.3)
Primary school	78,027 (23.2)	23,610 (30.8)	18,718 (25.7)	13,540 (23.2)	13,325 (20.9)	8834 (13.8)
Technical school	76,514 (22.8)	17,492 (22.8)	16,067 (22.1)	12,794 (21.9)	14,577 (22.8)	15,584 (24.3)
Secondary school	71,717 (21.4)	15,620 (20.3)	16,765 (23.1)	13,503 (23.1)	13,999 (21.9)	11,830 (18.5)
Longer education (Inc. university)	88,179 (26.3)	14,483 (18.9)	16,694 (23.0)	15,027 (25.7)	18,276 (28.6)	23,699 (37.0)
**BMI (SD) (kg/m**^**2**^**)**	25.3 (4.2)	26.0 (4.3)	25.5 (4.3)	25.3 (4.2)	25.1 (4.2)	24.4 (4.0)
**Smoking status (%)**						
Never	168,997 (49.5)	34,845 (44.7)	35,918 (48.5)	29,888 (50.3)	33,371 (51.4)	34,975 (53.7)
Former	94,690 (27.7)	19,357 (24.8)	19,552 (26.4)	16,623 (28.0)	18,953 (29.2)	20,205 (31.0)
Current	71,270 (20.9)	22,206 (28.5)	16,988 (22.9)	11,674 (19.6)	11,426 (17.6)	8976 (13.8)
**Alcohol intake (SD) (g/day)**	13.4 (18.0)	16.4 (20.9)	14.2 (18.7)	13.2 (17.4)	12.2 (16.5)	10.5 (14.6)
**Physical activity index**						
Inactive	59,201 (17.3)	12,221 (15.7)	12,394 (16.7)	10,428 (17.5)	11,560 (17.8)	12,598 (19.4)
Moderately inactive	120,386 (35.2)	25,570 (32.8)	26,485 (35.8)	21,712 (36.5)	23,981 (36.9)	22,638 (34.8)
Moderately active	122,917 (36.0)	28,608 (36.7)	26,451 (35.7)	21,167 (35.6)	23,191 (35.7)	23,500 (36.1)
Active	32,013 (9.4)	9331 (12.0)	7032 (9.5)	4995 (8.4)	5173 (8.0)	5482 (8.4)
**Total sugar (SD) (g/day)**	107.5 (45.1)	120.3 (48.1)	109.7 (44.5)	104.1 (43.2)	100.2 (42.4)	100.2 (42.9)
**Total energy (SD) (KJ/day)**	8878 (2577)	10,690 (25,98)	9388 (2351)	8631 (2209)	7974 (2096)	7255 (1983)
**Mean Healthy PDI (SD)**	51.6 (6.9)	42.8 (3.0)	48.6 (1.1)	52.0 (0.8)	55.4 (1.1)	61.7 (3.6)

PDI, plant-based diet index; BMI, body mass index; SD, standard deviation.

a Mean (SD) values and percentages are reported for continuous and categorical variables, respectively.

b The sex information was ascertained by self-reported information of participants at baseline recruitment centers.

We did not detect evidence of nonlinearity in the associations between the PDIs and risk of IBD in the UK Biobank study or the EPIC cohort (*P* for non-linear association >0.05) (Figure S3). For associations between individual food groups and incident IBD (Figure S4), the meta-analysis of results from the two cohorts showed that intake of sweets (HR 1.08, 95% CI 1.03–1.13, *P* = 0.001) and potatoes (HR 1.08, 95% CI 1.03–1.13, *P* = 0.001) was positively associated with IBD incidence, whereas fruit (HR 0.95, 95% CI 0.91–0.99, *P* = 0.022) and whole grain (HR 0.93, 95% CI 0.89–0.97, *P* = 0.002) intakes were inversely associated with IBD incidence.

### PDI and prognosis of IBD

**Fig. 4:**
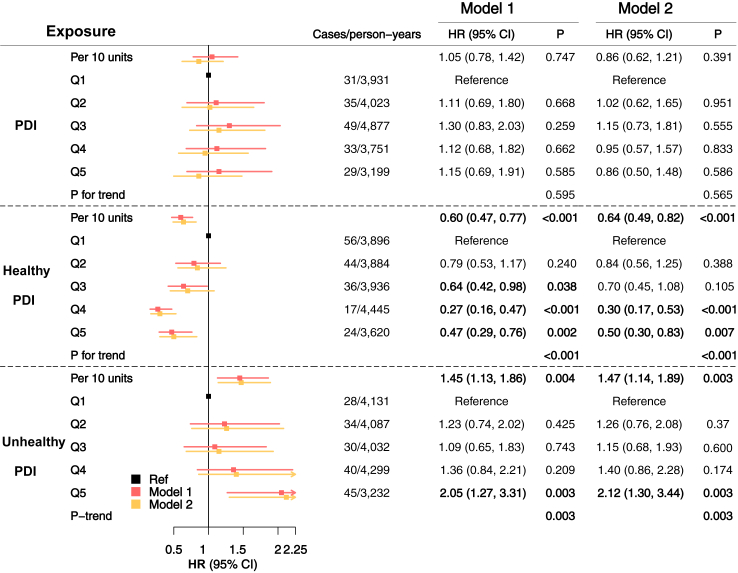
**Associations between the plant-based diet indexes and risk of related surgery among individuals) with IBD (n = 2133) in UK Biobank.** Model 1 was adjusted for age and sex, while Model 2 was additionally adjusted for Townsend deprivation index, education, ethnicity, body mass index (BMI), smoking status, alcohol consumption, exercise time, total energy intake, and total sugar intake. In this sample, the 20%, 40%, 60% and 80% percentile of PDIs were used as cutoff values as follow: PDI 46.6, 49.3, 52.0, 55.0; healthy PDI 50.7, 54.0, 57.0, 61.0; unhealthy PDI: 52.5, 56.0, 59.5, 63.0. Numbers in bold indicates significant associations. PDI, plant-based diet index; HR, hazard ratio; CI, confidence interval; EPIC, European Prospective Investigation into Cancer, and Nutrition.

The analysis of PDIs in relation to the risk of IBD comorbidities and outcome included 2133 individuals with IBD (631 CD, 1487 UC, and 15 unspecific types of IBD) from UK Biobank. Baseline characteristics are shown in Table S2. We documented 177 incident IBD-related surgery events (19,781 person-years), 142 cardiovascular disease (15,933 person-years), 119 diabetes mellitus (22,473 person-years), and 145 death events (20,727 person-years) during the follow-up of each outcome of interest. We found significant associations of the healthy and unhealthy PDIs with the risk of IBD-related surgery. Specifically, the HRs of risk of IBD-related surgery in the highest quintile of healthy PDI and unhealthy PDI compared with the lowest quintile were 0.50 (95% CI 0.30–0.83; *P* for trend = 0.0001) and 2.12 (95% CI 1.30–3.44; *P* for trend = 0.003), respectively (Fig. 4 and Figure S5). Similar associations were observed when separately examining the associations in participants of CD and UC (Table S3). We did not observe significant associations between PDIs and risk of cardiovascular disease, diabetes mellitus or mortality.

### Genetic susceptibility analysis

When considering the potential diet–genetic interaction, most associations between PDIs and incident IBD were consistent across individuals with different genetic susceptibility (Figure S6). However, most of the significant associations were observed in participants with moderate or high genetic risk with stronger effect estimates. The HRs of per 10 units increment in healthy PDIs [95% CI] for IBD were 1.01 (0.79–1.29), 0.71 (0.57–0.88), and 0.79 (0.65–0.95) among individuals with low, moderate, and high genetic risk of IBD, respectively (*P*-interaction = 0.079).

### Mediation analysis

For the significant associations identified in the primary and secondary analyses, we found the associations of the healthy and unhealthy PDIs with incident IBD and IBD-related surgeries were partially mediated by CRP levels (proportion 3.9–11.6%) and INFLA-score (8.9–22.3%), although clearly other mechanism are at play to explain the inverse proportion, which needs further research (Figure S7).

### Sensitivity analysis

In sensitivity analyses, we found an inverse association between a healthy omnivorous diet and incident IBD (HR _comparing extreme quintiles_ 0.71 95% 0.56–0.89, *P*-trend = 0.0005), and the effect of the healthy omnivorous diet slightly attenuated after adjustment of dietary fibre (Table S4). The associations between PDIs with the incidence and prognosis of IBD were consistent in the analysis among participants with at least two dietary recalls (HR of per 10 units increment in healthy PDIs [95% CI] were 0.82 [0.70–0.97] for incident IBD and 0.60 [0.42–0.86] for IBD-related surgery, Figure S8) or further adjusted for ultra-processed food intake (HR of per 10 units increment in healthy PDIs [95% CI] were 0.80 [0.71–0.90] for incident IBD and 0.64 [0.50–0.83] for IBD-related surgery), so that the main conclusion did not change after adjusting for ultra-processed food intake (Figure S9). The associations between healthy PDIs and risk of IBD-related surgery were consistent after adjustment for IBD-related medication (Figure S10). And we also observed consistent associations between healthy PDIs and risk of IBD incidence and related surgery when applying competing risk model (Figure S11).

## Discussion

In two large cohorts covering more than 500,000 participants across 8 nations, we found that a healthy PDI was inversely associated with IBD incidence and IBD-related surgery, whereas an unhealthy PDI was positively associated with the same outcomes. These associations were partially mediated by inflammation and may be greater for participants with moderate or high genetic risk to develop IBD.

Our results are partly in line with a previous prospective cohort study of 83,147 participants from two Swedish cohorts showed that healthy PDI was associated with a lower risk of older-onset CD but not UC (HR _comparing extreme quartiles_ 0.84, 95% CI 0.63–1.18).^37^ In our study, we were able to replicate the finding in CD, while also reporting a link for UC in the same direction, probably due to our larger sample size. We were also able to further expand the findings by showing an inverse association when adhering to an unhealthy plant-based diet. When further exploring the association of PDI with the disease course of IBD, we found both a healthy and an unhealthy PDIs were both, inversely associated with the risk of IBD-related surgery but not with cardiovascular diseases, diabetes, or all-cause mortality. IBD-related surgery is a novel finding, that is often not taken into account in other cohort studies, which we were able to do due to the long follow-up and the inclusion of IBD-patients at baseline in the UK biobank. Importantly, our finding that not all plant-based diets equal a beneficial outcome in IBD, is novel and of interest given the current research interest in vegan diet, *lacto-ovo* vegetarian diet, or more flexible semi-vegetarian diets.^5^^,^^27^^,^^38^

Our findings could be explained by the addition of beneficial components that come with a healthy plant-based dietary pattern, including a high consumption of fruits, vegetables, legumes, and dietary fiber, which were proposed to protect against development^39^ and disease course^7^^,^^40^ of IBD. Higher adherence to a plant-based diet also brings more intake of plant phytochemicals. Numerous experiments have shown that the plant-derived phytochemicals could reduce permeability, ease oxidative stress, and reduce pro-inflammatory cytokines secretion.^41^ Lastly, previous studies demonstrated inverse associations between dietary fibre intake and risk of relapse and related surgery risk among individuals with IBD, providing indirect evidence to support our observed results.^42^ However, our results showed that the association between a healthy omnivorous diet (which allows the intake of dairy, eggs, and fish in the healthy PDI algorithm) and IBD slightly increased the confidence interval after adjusting for dietary fiber, indicating that the association between a healthy plant-based diet and IBD may not be limited solely to the increased intake of dietary fiber.

In our mediation analysis, only up to 22% of the associations could be explained by inflammation. This may partly be due to the relatively low CRP and INFLA scores observed in the participants. Additionally, other inflammatory markers were not assessed, which might have limited our ability to fully capture inflammatory pathways. Furthermore, it is possible the incidence and prognosis of IBD could be affected by plant-based diet through alternative mechanisms beyond inflammation, which warrants further investigation.

Certain food components that can be found in plant-based foods might induce intestinal inflammation. For example, artificial sweeteners and dietary emulsifiers have been shown to induce or deteriorate experimental gut inflammation by microbiota-dependent and independent pathways in the genetically susceptible host.^43^, ^44^, ^45^ In addition, previous work has revealed positive associations of ultra-processed food with both incidence and disease course of IBD.^31^ In our sensitivity analyses, we observed a consistently protective potential of a plant-based diet, independent of ultra-processed food intake.

The analysis for associations between individual food components and IBD (Figure S4) revealed that most of the food groups did not reach statistically significant associations with IBD, despite the consistent direction of the associations. In the case of potatoes, for example, our study found a positive association between potatoes and the development of IBD. This was inconsistent with a previous study demonstrating a inverse associations between potato intake and IBD disease severity.^46^ Clearly, further research is necessary to distinguish the ‘healthy’ and ‘unhealthy’ components of any plant-based diet and how to translate and balance this into clinical dietary management in IBD prevention and treatment. The health effects of fried potatoes and potato snacks versus boiled potatoes may be quite different from an ultra-processed food perspective; however, epidemiological studies have similarly found that potatoes regardless of preparation method, as part of a “Westernized” diet, are associated with weight gain and an increased risk of T2DM.^47^

In the main analysis, the incidence of IBD among UKB participants was highest in the fourth rather than the fifth quartile of the PDI (which assigns positive scores to all plant-based diets) score, also suggesting that it is also important to avoid unhealthy plant foods while practicing a plant-based diet. Specialized dietary counselling can also help ensure that plant-based diets emphasize whole, minimally processed foods, such as fruits, vegetables, legumes, nuts, and whole grains, while reducing reliance on ultra-processed plant-derived products. Such counselling is particularly relevant in the context of changing food environments, where the availability of ultra-processed vegetarian and vegan options has increased substantially.^48^ Personalized guidance can further address individual needs, such as nutrient adequacy, energy balance, and avoidance of IBD-specific dietary triggers, thereby optimizing dietary quality and supporting long-term health outcomes.

The earlier data collection in EPIC coincided with a time when the availability of highly processed plant-based foods was more limited, potentially resulting in a smaller magnitude of HRs compared to more recent UK Biobank data. This highlights the growing complexity of nutritional epidemiology and the importance of distinguishing truly health-promoting plant-based foods from processed alternatives, even within the “plant-based” categorization.^12^^,^^15^^,^^48^ The rapid change of dietary habits and food compositions over the past two decades also warrants further exploration. The global rise in plant-based eating patterns has been accompanied by the proliferation of processed plant-based products, such as meat substitutes and refined plant oils, which may dilute the health benefits of traditional plant-based diets if not properly accounted for.^48^ This underscores the need for contemporary datasets that reflect current dietary trends and food composition.

Nonetheless, differences in cohort characteristics, including regions (multi-country EPIC versus UK-specific UK Biobank), data collection periods (1990s for EPIC versus 2009–2010 for UK Biobank), and dietary assessment methods (FFQ versus 24-h dietary recall) between the cohorts could introduce misclassification; despite this, meta-analyses did not reveal substantial heterogeneity in the data from the two studies. Discrepancies in their sensitivity to detect certain dietary patterns or transient dietary changes could impact the estimation of PDI scores. This might also be the reason for slight variations in effect sizes between the cohorts, particularly in the extreme quintiles of unhealthy PDI. Nonetheless, the inclusion of two cohorts with differing methodologies and timeframes should strengthen the external validity of our findings. Prospective studies incorporating standardized dietary tools and longitudinal dietary tracking would help resolve uncertainties arising from temporal and methodological variations.

There are also several other limitations in our study. Our study remains of observational nature, limiting the determination of potentially causal associations because residual confounding may remain; to address this, we considered a comprehensive list of covariates. To reduce reverse causation, we excluded participants with IBD diagnosed in the first year of follow-up. Next, measurement error may exist in dietary assessment, although multiple recalls of food records and questionnaires were used for analyses to construct the PDI. We also excluded participants with only one dietary recall to minimise measurement error in a sensitivity analysis. Furthermore, participants included in the current analyses were predominantly European and white adults, limiting the generalization of the findings to other populations such as children and different ethnic groups. Finally, while we explored clinically relevant outcomes in patients with IBD, such as surgery, cardiometabolic comorbidities, and mortality, data availability limited our ability to investigate other important outcomes, such as disease activity, relapse, and quality of life. These outcomes are critical for a more comprehensive understanding of the impact of plant-based diets on the natural course of IBD. Future research is needed to address these additional endpoints and to further explore the mechanistic pathways linking dietary patterns with IBD prognosis.

### Conclusion

Our findings provide evidence for the protective effect of healthy plant-based diets on IBD incidence and progression in two European cohort studies, while an adverse association was found for an unhealthy plant-based diet. The associations between the healthy and unhealthy PDIs and IBD were partially mediated by inflammation and may be greater for participants with moderate or high genetic risk to develop IBD. These findings support the current paradigm that plant-based foods should be recommended to all patients with IBD yet underline the possible need for specialised dietetic counselling to ensure the overall quality of the diet. Further mechanistic work is needed to disentangle which factors drive the opposing results to allow for targeted dietary modification.

## Contributors

Study conceptualization was contributed by J.C., Y.S., L.D., J.W., S.Y., Y.H., T.T., A.C., N.P., A.M., C.D., S.L., A.W., J.L., K.T., E.G., J.S., S.C., and X.L. Methodology was developed by J.C., Y.S., L.D., J.W., T.T., A.C., N.P., A.M., C.D., S.L., A.W., J.L., K.T., E.G., J.S., S.C., and X.L. Manuscript drafting and review were carried out by J.C., Y.S., L.D., J.W., S.Y., Y.H., T.T., A.C., N.P., A.M., C.D., S.L., A.W., J.L., K.T., E.G., J.S., and X.L. Manuscript reviewing and editing were led by X.W. and E.T., with contributions from all authors. Data acquisition was carried out by S.C. Project administration was led by X.L.

Data verification was performed by J.C., Y.S., L.D., and X.L. Formal analysis was conducted by Y.S., L.D., and J.C. All authors had full access to the data and were responsible for the decision to submit the paper for publication. All authors have read and approved the final version of the manuscript.

## Data sharing statement

The datasets analysed during the current study are available in public, open access repository (https://www.ukbiobank.ac.uk/and https://epic.iarc.fr/).

## Declaration of interests

JFL has coordinated an unrelated study on behalf of the Swedish IBD quality register (SWIBREG). That study received funding from Janssen corporation. JFL has also received financial support from MSD developing a paper reviewing national healthcare registers in China. JFL has an ongoing research collaboration on celiac disease with Takeda.

No financial disclosures were reported by the other authors of this paper.

Where authors are identified as personnel of the International Agency for Research on Cancer/World Health Organization, the authors alone are responsible for the views expressed in this article and they do not necessarily represent the decisions, policy or views of the International Agency for Research on Cancer/World Health Organization.

## Appendix A Supplementary data

Supplementary Figures, Tables and EPIC Investigator
