# Supplementary material for: Composition of plant-based diets and the incidence and prognosis of inflammatory bowel disease: a multinational retrospective cohort study
Source: Lancet Reg Health Eur. 2025 Mar 14;52:101264. doi: 10.1016/j.lanepe.2025.101264 (PMC11957509; doi:10.1016/j.lanepe.2025.101264)
Supplement: Supplementary Figures, Tables and EPIC Investigator [file mmc1.docx]

**SUPPLEMENTARY MATERIALS**

**Composition of plant-based diets and the incidence and prognosis of inflammatory bowel disease: a** **multinational retrospective cohort study**

Content

[Supplementary Method 3](#_Toc191173858)

[Table S1 Definitions of IBD and IBD-related clinical outcomes 10](#_Toc191173859)

[Table S2 Characteristics of participants with IBD by healthy plant-based diet index quintiles in the UK Biobank 11](#_Toc191173860)

[Table S3 Associations of plant-based diet indexes with clinical outcomes among individuals with Crohn’s disease and ulcerative colitis in the UK Biobank 12](#_Toc191173861)

[Table S4 Associations of healthy omnivorous diet with incident inflammatory bowel disease in the UK Biobank 15](#_Toc191173862)

[Figure S1 Associations between the plant-based diet indexes and incident Crohn’s disease in UK Biobank (n=187,888), EPIC cohort (n=341,539), and the meta-analysis of the results from the two cohorts (n=529,427). 16](#_Toc191173863)

[Figure S2. Associations between the plant-based diet indexes and incident ulcerative colitis in UK Biobank (n=187,888), EPIC cohort (n=341,539), and the meta-analysis of the results from the two cohorts (n=529,427). 17](#_Toc191173864)

[Figure S3 Non-linear relationship of the plant-based diet indexes with risk of incident inflammatory bowel disease in the UK Biobank (a-c) and EPIC cohort (d-f). 18](#_Toc191173865)

[Figure S4. Associations between the individual food categories and incident inflammatory bowel disease in UK Biobank (n=187,888), EPIC cohort (n=341,539), and the meta-analysis of the results from the two cohorts (n=529,427). 19](#_Toc191173866)

[Figure S5 Associations of plant-based diet indexes with clinical outcomes (except for IBD-related surgery) among individuals with IBD in the UK Biobank. 20](#_Toc191173867)

[Figure S6 Associations of plant-based diet indexes with incident inflammatory bowel disease stratified by genetic risk categories (by tertiles of Polygenic Risk Score) in the UK Biobank. 21](#_Toc191173868)

[Figure S7 mediation analysis for associations between healthy and unhealthy PDI and incident IBD and IBD-related surgery. The magnitude of the coefficients of the direct and indirect effects is 10^-4^. 22](#_Toc191173869)

[Figure S8 Associations of plant-based diet indexes with incident IBD (among individuals free of IBD at bassline) and clinical outcomes (among individuals with IBD) in the UK Biobank participants with at least two dietary recalls.. 23](#_Toc191173870)

[Figure S9 Associations of plant-based diet indexes with incident IBD (among individuals free of IBD at bassline) and clinical outcomes (among individuals with IBD) in the UK Biobank participants further adjusted for intake of ultra-processed food.. 24](#_Toc191173871)

[Figure S10 Associations of plant-based diet indexes with clinical outcomes (among individuals with IBD) in the UK Biobank participants further adjusted for IBD-related medication. 25](#_Toc191173872)

[Figure S11 Associations of plant-based diet indexes with incident IBD (among individuals free of IBD at bassline) and clinical outcomes (among individuals with IBD) in the UK Biobank participants treating death as competing risk events. 26](#_Toc191173873)

[Investigator list of EPIC 27](#_Toc191173874)

# **Supplementary Method**

***Exposure process in UKB and EPIC cohort***

In the UK Biobank, dietary information was collected by a Web-based 24-hour dietary recall (WebQ) administered in five rounds between 2009 and 2012, reporting daily intake of over 200 common foods and 30 beverages. The WebQ is in good agreement with long-term consumption and frequency of food groups collected by baseline food frequency questionnaires[1]. Compared to interviewer-administered 24-hour recall completed on the same day, Spearman correlation coefficients calculated from the WebQ ranged from 0.5 and 0.9 (mean 0.6) for most nutrients[2].

In the EPIC study, dietary information was assessed using validated country-specific food frequency questionnaires (FFQs) at baseline recruitment, evaluating the regular diet covering 98 to 260 food items during the preceding 12 months[3]. Reported food items were categorized according to the harmonized food categories common to each questionnaire. In all recruitment centres, the FFQs were validated using 24-hour recall questionnaires, with Spearman correlation coefficients ranging from 0.37 to 0.79 for food groups.[4]

Three versions of the PDI (overall PDI, healthy PDI, and unhealthy PDI) were constructed by scoring the intake of three broad food groups (healthy plant foods, unhealthy plant foods, and animal foods) comprising 17 food groups[5,6]. The overall PDI assigned positive scores to all plant foods. The healthy PDI assigned positive scores to healthy plant foods (whole grains, fruits, vegetables, nuts, legumes and vegetarian protein alternatives, tea and coffee) and reverse scores to unhealthy plant foods (refined grains, potatoes/fries, fruit juices, sugar-sweetened beverages, sweets and desserts). The unhealthy PDI assigned positive scores to unhealthy plant foods and reverse scores to healthy plant foods. In all three PDIs, animal foods (animal fat, dairy, egg, fish or seafood, meat, and miscellaneous animal-based foods) were given reverse scores. For positive scores, the highest intake quintile scored 5 points and the lowest intake quintile scored 1 point, and vice versa. These indicators could present incremental dietary changes instead of defining the plant-based diet as vegetarian diets and dichotomizing study populations by consumption of animal foods. In addition, we also created a ‘healthy omnivorous diet’ based on the healthy PDI by assigning certain typically assumed healthy animal food (dairy, eggs, and fish or seafood) positive scores for sensitivity analysis[7]. The common food items used to calculate the dietary indexes were presented in the following table

**Common food items used to calculation of PDIs in the UKB and EPIC**

| **Food categories** | **Food groups** | **Common food items in UKB** | **Common food items in EPIC** | **PDI** | **Healthy PDI** | **Unhealthy PDI** | **healthy omnivorous diet** |
| --- | --- | --- | --- | --- | --- | --- | --- |
| **Healthy Plant food** | **Fruits** | Prune, Apple, Banana, Berry, Cherry, Grapefruit | Citrus fruit, apple and pear, grape, stone fruit, berries | Positive | Positive | Negative | Positive |
|  | **Vegetables** | Vegetable salad, Avocado, Beetroot, Broccoli, Butternut squash, | Leafy vegetables, fruiting vegetables, root vegetables, cabbages, mixed salad | Positive | Positive | Negative | Positive |
|  | **Whole grains** | Whole-wheat cereal, oat cereal, bran cereal, wholemeal pasta, brown rice | Non-white bread, non-white crispbread/rusks | Positive | Positive | Negative | Positive |
|  | **Nuts** | nuts, peanut, seeds | Tree nuts, peanut, seeds, chestnuts | Positive | Positive | Negative | Positive |
|  | **Legumes** | Pulses, bean, pea, tofu | Soya products | Positive | Positive | Negative | Positive |
|  | **Vegetarian protein alternatives** | Vegetarian burgers and Quorn | N/A | Positive | Positive | Negative | Positive |
|  | **Tea/coffee** | Coffee, tea, herbal tea | Coffee, tea, herbal tea, chicory/substitutes | Positive | Positive | Negative | Positive |
| **Unhealthy plant food** | **Refined grains** | Sweetened cereal, plain cereal, white pasta, white rice, sushi intake | Pasta, rice, white bread, crispbread, crackers, | Positive | Negative | Positive | Negative |
|  | **Potatoes** | Mashed/boiled/fried potatoes | Potatoes, other tubes | Positive | Negative | Positive | Negative |
|  | **Fruit juices** | Orange/Grapefruit/Pure fruit/vegetable juice | Citrus and vegetable juices | Positive | Negative | Positive | Negative |
|  | **Sugar-sweetened beverages** | Fizzy drink, squash intake | Carbonated/soft/isotonic drinks | Positive | Negative | Positive | Negative |
|  | **Sweets and desserts** | Pastry, croissant, scone, pudding, fruitcake, cake intake | Honey, jam and syrup, chocolate, candy bars, paste, confetti, cakes, sweet pies | Positive | Negative | Positive | Negative |
| **Animal food** | **Animal fat** | Animal fat | Butte/Margarine, deep frying fats, Other | Negative | Negative | Negative | Negative |
|  | **Dairy** | Cheese, milk, yogurt, dairy smoothie | Milk, yoghurt, cheese, curd cream | Negative | Negative | Negative | Positive |
|  | **Egg** | Whole egg, omelette, scotch egg, other | Eggs | Negative | Negative | Negative | Positive |
|  | **Fish or seafood** | Oily fish, white fish, Crab/lobster, shellfish, prawns | Lean fish, white fish, related fish product, roe | Negative | Negative | Negative | Positive |
|  | **Meat** | Beef, sausage, beef, poultry, ham | Beef, veal, pork, mutton/lamb, poultry rabbit, processed meats | Negative | Negative | Negative | Negative |
|  | **Miscellaneous animal-based foods** | Pizza, Indian Snacks | Hamburger, meatballs | Negative | Negative | Negative | Negative |

**Reference**

[1] Bradbury, K.E.; Young, H.J.; Guo, W.; Key, T.J. Dietary assessment in UK Biobank: an evaluation of the performance of the touchscreen dietary questionnaire. Journal of nutritional science 2018, 7, e6, doi:10.1017/jns.2017.66.

[2] Liu, B.; Young, H.; Crowe, F.L.; Benson, V.S.; Spencer, E.A.; Key, T.J.; Appleby, P.N.; Beral, V. Development and evaluation of the Oxford WebQ, a low-cost, web-based method for assessment of previous 24 h dietary intakes in large-scale prospective studies. Public Health Nutr 2011, 14, 1998-2005, doi:10.1017/s1368980011000942.

[3] Riboli, E.; Hunt, K.J.; Slimani, N.; Ferrari, P.; Norat, T.; Fahey, M.; Charrondière, U.R.; Hémon, B.; Casagrande, C.; Vignat, J.; et al. European Prospective Investigation into Cancer and Nutrition (EPIC): study populations and data collection. Public Health Nutr 2002, 5, 1113-1124, doi:10.1079/phn2002394.

[4] Kaaks, R.; Slimani, N.; Riboli, E. Pilot phase studies on the accuracy of dietary intake measurements in the EPIC project: overall evaluation of results. European Prospective Investigation into Cancer and Nutrition. Int J Epidemiol 1997, 26 Suppl 1, S26-36, doi:10.1093/ije/26.suppl_1.s26.

[5] Heianza, Y.; Zhou, T.; Sun, D.; Hu, F.B.; Qi, L. Healthful plant-based dietary patterns, genetic risk of obesity, and cardiovascular risk in the UK biobank study. Clin Nutr 2021, 40, 4694-4701, doi:10.1016/j.clnu.2021.06.018.

[6] Satija, A.; Bhupathiraju, S.N.; Rimm, E.B.; Spiegelman, D.; Chiuve, S.E.; Borgi, L.; Willett, W.C.; Manson, J.E.; Sun, Q.; Hu, F.B. Plant-Based Dietary Patterns and Incidence of Type 2 Diabetes in US Men and Women: Results from Three Prospective Cohort Studies. PLoS Med 2016, 13, e1002039, doi:10.1371/journal.pmed.1002039.

[7]Satija, A.; Bhupathiraju, S.N.; Spiegelman, D.; Chiuve, S.E.; Manson, J.E.; Willett, W.; Rexrode, K.M.; Rimm, E.B.; Hu, F.B. Healthful and Unhealthful Plant-Based Diets and the Risk of Coronary Heart Disease in U.S. Adults. J Am Coll Cardiol 2017, 70, 411-422, doi:10.1016/j.jacc.2017.05.047.

***Ascertainment of outcome and follow-up***

In the UK Biobank, the outcome events were ascertained by external linkage to national hospital inpatient records, primary care data, and death registry. In the EPIC study, participants who developed incident IBD were identified either by self-administered follow-up questionnaires with medical records review by 1-2 physicians or national/regional registries, depending on the centre. Person-years were calculated from the date the last WebQ was completed to the date of the first occurrence of any of the outcomes of interest, date of death, date of loss to follow-up, or the end of follow-up, whichever came first.

***Definition of INFLA-score***

We used the following blood-derived markers measured at baseline recruitment to calculate an INFLA-score: CRP, white blood cell count, platelet count, and neutrophil-to-lymphocyte ratio. Each indicator was divided into deciles and assigned a score of +1 to +4 for the highest deciles (7th to 10th) respectively; a score of -4 to -1 for the lowest (1st to 4th); and 0 for the remaining deciles. The scores for the four indicators were summed to generate an INFLA score ranging from -16 to +16, with higher scores indicating increased inflammation [1].

Reference

[1] Bonaccio M, Di Castelnuovo A, Pounis G, et al. A score of low-grade inflammation and risk of mortality: prospective findings from the Moli-sani study. Haematologica 2016;101(11):1434-41. doi: 10.3324/haematol.2016.144055 [published Online First: 2016/11/02]

***Genetic risk profiling***

We first constructed a PRS using the common genetic variants that were identified to be strongly associated with CD and UC (*P* < 5 × 10^−8^) from a genome-wide association meta-analysis of up to 86,640 individuals of European ancestry [1]. After removing genetic variants in linkage disequilibrium, 72, 51 and 30 independent single-nucleotide polymorphisms (SNP) (r^2^ < 0.001) were used to calculate the PRS of IBD, CD, and UC, respectively. Polygenic risk scores were constructed for each participant by summing up the number of risk-increasing alleles for each SNP weighted by effect size on genetic liability to IBD, CD or UC. Genome-wide genetic data of UK Biobank participants were tested by UK BiLEVE Axiom array or UK Biobank Axiom array. Imputation was performed using computationally efficient methods combined with the Haplotype Reference Consortium and UK10K haplotype resources, providing additional coverage for the genome.

Reference

[1] Liu JZ, van Sommeren S, Huang H, et al. Association analyses identify 38 susceptibility loci for inflammatory bowel disease and highlight shared genetic risk across populations. Nat Genet 2015;47(9):979–86.

| **Outcome** | **SNP** | **EA** | **NEA** | **Beta** | **SE** | ***P*-value** |
| --- | --- | --- | --- | --- | --- | --- |
| IBD | rs3766606 | A | C | -0.117 | 0.016 | 1.07×10^-09 |
| IBD | rs3806308 | A | G | -0.094 | 0.011 | 2.97×10^-08 |
| IBD | rs6426833 | G | A | -0.128 | 0.012 | 1.39×10^-16 |
| IBD | rs12568930 | G | A | -0.083 | 0.015 | 1.67×10^-09 |
| IBD | rs7517847 | C | A | -0.248 | 0.013 | 1.38×10^-40 |
| IBD | rs1801274 | G | A | -0.128 | 0.012 | 2.66×10^-13 |
| IBD | rs7554511 | A | C | -0.163 | 0.013 | 7.35×10^-15 |
| IBD | rs3024505 | A | G | 0.199 | 0.011 | 1.35×10^-21 |
| IBD | rs34856868 | A | G | -0.195 | 0.043 | 9.8×10^-09 |
| IBD | rs6025 | A | G | -0.178 | 0.040 | 2.51×10^-08 |
| IBD | rs10798069 | A | C | -0.070 | 0.013 | 4.25×10^-09 |
| IBD | rs7555082 | A | G | 0.124 | 0.017 | 1.47×10^-10 |
| IBD | rs7608910 | G | A | 0.122 | 0.009 | 6.98×10^-16 |
| IBD | rs10185424 | A | C | 0.086 | 0.009 | 5.81×10^-09 |
| IBD | rs12994997 | G | A | -0.139 | 0.012 | 4.09×10^-18 |
| IBD | rs3749171 | A | G | 0.113 | 0.012 | 2.67×10^-09 |
| IBD | rs11681525 | C | G | -0.146 | 0.026 | 4.08×10^-11 |
| IBD | rs4664304 | A | G | 0.056 | 0.010 | 2.61×10^-08 |
| IBD | rs35320439 | G | A | 0.084 | 0.013 | 9.89×10^-10 |
| IBD | rs9868809 | A | G | 0.140 | 0.014 | 5.53×10^-11 |
| IBD | rs113010081 | G | A | 0.127 | 0.019 | 9.02×10^-10 |
| IBD | rs4692386 | A | G | -0.058 | 0.011 | 1.21×10^-08 |
| IBD | rs2189234 | A | C | 0.081 | 0.012 | 1.95×10^-10 |
| IBD | rs11742570 | A | G | -0.174 | 0.012 | 2.88×10^-27 |
| IBD | rs17622378 | G | A | 0.140 | 0.009 | 1.3×10^-15 |
| IBD | rs254560 | A | G | 0.058 | 0.010 | 6.16×10^-09 |
| IBD | rs11741861 | G | A | 0.199 | 0.014 | 2.39×10^-10 |
| IBD | rs6556412 | A | G | 0.122 | 0.010 | 4.81×10^-14 |
| IBD | rs395157 | A | G | 0.091 | 0.009 | 2.22×10^-20 |
| IBD | rs4703855 | A | G | -0.071 | 0.012 | 7.16×10^-11 |
| IBD | rs17119 | G | A | -0.083 | 0.013 | 5.37×10^-09 |
| IBD | rs6908425 | A | G | -0.083 | 0.013 | 1.11×10^-08 |
| IBD | rs7746082 | C | G | 0.104 | 0.010 | 1.25×10^-08 |
| IBD | rs3851228 | A | T | 0.148 | 0.017 | 3.97×10^-08 |
| IBD | rs6920220 | A | G | 0.095 | 0.011 | 4.69×10^-08 |
| IBD | rs1819333 | C | A | -0.083 | 0.011 | 8.21×10^-09 |
| IBD | rs7773324 | G | A | -0.079 | 0.014 | 1.06×10^-09 |
| IBD | rs13204048 | G | A | -0.068 | 0.013 | 2.89×10^-08 |
| IBD | rs7758080 | G | A | 0.076 | 0.012 | 7.27×10^-09 |
| IBD | rs4380874 | A | G | 0.077 | 0.009 | 2.62×10^-13 |
| IBD | rs1077773 | G | A | -0.072 | 0.014 | 5.96×10^-09 |
| IBD | rs2538470 | A | G | 0.068 | 0.010 | 3×10^-11 |
| IBD | rs921720 | A | G | -0.083 | 0.011 | 5.13×10^-09 |
| IBD | rs4246905 | A | G | -0.128 | 0.013 | 1.42×10^-16 |
| IBD | rs10781499 | A | G | 0.157 | 0.009 | 4.16×10^-25 |
| IBD | rs11010067 | G | C | 0.104 | 0.009 | 3.14×10^-11 |
| IBD | rs10761659 | A | G | -0.151 | 0.012 | 4.07×10^-21 |
| IBD | rs4409764 | A | C | 0.166 | 0.009 | 2.64×10^-25 |
| IBD | rs2155219 | A | C | 0.148 | 0.009 | 2.83×10^-17 |
| IBD | rs12422544 | G | A | 0.300 | 0.024 | 2.26×10^-13 |
| IBD | rs7134472 | A | G | 0.104 | 0.009 | 7.11×10^-15 |
| IBD | rs7954567 | A | G | 0.082 | 0.013 | 1.3×10^-09 |
| IBD | rs653178 | G | A | 0.056 | 0.009 | 1.11×10^-08 |
| IBD | rs9525625 | A | G | 0.074 | 0.011 | 1.41×10^-09 |
| IBD | rs17293632 | A | G | 0.104 | 0.011 | 5.71×10^-14 |
| IBD | rs26528 | G | A | 0.095 | 0.009 | 1.5×10^-09 |
| IBD | rs3091315 | G | A | -0.105 | 0.012 | 4.84×10^-09 |
| IBD | rs12946510 | A | G | 0.131 | 0.009 | 1.35×10^-15 |
| IBD | rs12942547 | G | A | -0.094 | 0.011 | 2.77×10^-11 |
| IBD | rs3853824 | A | G | -0.081 | 0.014 | 1.17×10^-10 |
| IBD | rs17736589 | G | A | 0.082 | 0.014 | 4.34×10^-08 |
| IBD | rs1893217 | G | A | 0.140 | 0.011 | 4.61×10^-11 |
| IBD | rs7236492 | A | G | -0.100 | 0.020 | 9.09×10^-09 |
| IBD | rs2024092 | A | G | 0.104 | 0.011 | 2.69×10^-11 |
| IBD | rs12720356 | C | A | 0.148 | 0.016 | 6.52×10^-11 |
| IBD | rs6062504 | A | G | -0.105 | 0.012 | 3.42×10^-15 |
| IBD | rs2823286 | A | G | -0.117 | 0.013 | 4.52×10^-11 |
| IBD | rs2836878 | A | G | -0.163 | 0.014 | 3.7×10^-22 |
| IBD | rs7282490 | G | A | 0.113 | 0.009 | 1.28×10^-17 |
| IBD | rs2256609 | G | A | 0.086 | 0.011 | 4.43×10^-10 |
| IBD | rs2413583 | A | G | -0.174 | 0.017 | 1.6×10^-12 |
| IBD | rs727563 | G | A | 0.092 | 0.013 | 1.88×10^-10 |
| CD | rs7517847 | C | A | -0.342 | 0.017 | 2.29×10^-46 |
| CD | rs7517810 | A | G | 0.131 | 0.012 | 1.11×10^-14 |
| CD | rs3024505 | A | G | 0.166 | 0.014 | 3.91×10^-09 |
| CD | rs10798069 | A | C | -0.073 | 0.011 | 4.25×10^-09 |
| CD | rs7555082 | A | G | 0.122 | 0.018 | 1.47×10^-10 |
| CD | rs10495903 | A | G | 0.122 | 0.015 | 3.3×10^-08 |
| CD | rs6708413 | G | A | 0.113 | 0.013 | 1.54×10^-10 |
| CD | rs6716753 | G | A | 0.131 | 0.013 | 1.87×10^-08 |
| CD | rs12994997 | G | A | -0.223 | 0.015 | 2.06×10^-37 |
| CD | rs11681525 | C | G | -0.151 | 0.024 | 4.08×10^-11 |
| CD | rs35320439 | G | A | 0.086 | 0.009 | 9.89×10^-10 |
| CD | rs3197999 | A | G | 0.157 | 0.011 | 9.13×10^-13 |
| CD | rs11742570 | A | G | -0.248 | 0.016 | 4.47×10^-34 |
| CD | rs1363907 | A | G | 0.104 | 0.012 | 1.46×10^-11 |
| CD | rs11743851 | G | A | 0.140 | 0.011 | 8.69×10^-12 |
| CD | rs11741861 | G | A | 0.285 | 0.015 | 4.6×10^-16 |
| CD | rs6556412 | A | G | 0.157 | 0.011 | 8.03×10^-15 |
| CD | rs6908425 | A | G | -0.105 | 0.017 | 2.42×10^-08 |
| CD | rs7746082 | C | G | 0.131 | 0.012 | 1.77×10^-08 |
| CD | rs1819333 | C | A | -0.117 | 0.014 | 9.3×10^-12 |
| CD | rs7773324 | G | A | -0.083 | 0.011 | 1.06×10^-09 |
| CD | rs13204048 | G | A | -0.073 | 0.011 | 2.89×10^-08 |
| CD | rs7758080 | G | A | 0.077 | 0.009 | 7.27×10^-09 |
| CD | rs1456896 | G | A | -0.094 | 0.014 | 2.9×10^-08 |
| CD | rs921720 | A | G | -0.117 | 0.014 | 6.4×10^-12 |
| CD | rs4246905 | A | G | -0.139 | 0.016 | 1.28×10^-14 |
| CD | rs10781499 | A | G | 0.166 | 0.010 | 1.03×10^-19 |
| CD | rs11010067 | G | C | 0.131 | 0.011 | 2.33×10^-09 |
| CD | rs10761659 | A | G | -0.186 | 0.015 | 3.42×10^-19 |
| CD | rs4409764 | A | C | 0.174 | 0.010 | 8.48×10^-19 |
| CD | rs2155219 | A | C | 0.174 | 0.010 | 6.51×10^-13 |
| CD | rs12422544 | G | A | 0.378 | 0.025 | 3.29×10^-12 |
| CD | rs7954567 | A | G | 0.086 | 0.009 | 1.3×10^-09 |
| CD | rs3764147 | G | A | 0.140 | 0.012 | 7.31×10^-09 |
| CD | rs9525625 | A | G | 0.077 | 0.009 | 1.41×10^-09 |
| CD | rs17293632 | A | G | 0.131 | 0.012 | 1.08×10^-12 |
| CD | rs26528 | G | A | 0.122 | 0.011 | 1.06×10^-08 |
| CD | rs3091315 | G | A | -0.139 | 0.016 | 9.52×10^-12 |
| CD | rs12946510 | A | G | 0.122 | 0.011 | 4.3×10^-08 |
| CD | rs3853824 | A | G | -0.083 | 0.011 | 1.17×10^-10 |
| CD | rs1893217 | G | A | 0.166 | 0.014 | 1.92×10^-12 |
| CD | rs7236492 | A | G | -0.094 | 0.022 | 9.09×10^-09 |
| CD | rs2024092 | A | G | 0.148 | 0.012 | 2.26×10^-11 |
| CD | rs11879191 | A | G | -0.139 | 0.019 | 1.66×10^-08 |
| CD | rs516246 | A | G | 0.113 | 0.011 | 1.21×10^-08 |
| CD | rs6062504 | A | G | -0.105 | 0.015 | 3.28×10^-10 |
| CD | rs2823286 | A | G | -0.139 | 0.015 | 1.24×10^-09 |
| CD | rs7282490 | G | A | 0.122 | 0.011 | 3.81×10^-13 |
| CD | rs2256609 | G | A | 0.104 | 0.014 | 8.02×10^-09 |
| CD | rs2413583 | A | G | -0.211 | 0.021 | 3.65×10^-10 |
| CD | rs727563 | G | A | 0.095 | 0.009 | 1.88×10^-10 |
| UC | rs6667605 | A | G | -0.083 | 0.014 | 1.4×10^-08 |
| UC | rs3806308 | A | G | -0.174 | 0.016 | 9.81×10^-15 |
| UC | rs6426833 | G | A | -0.236 | 0.017 | 4.86×10^-31 |
| UC | rs12568930 | G | A | -0.128 | 0.021 | 6.24×10^-11 |
| UC | rs7517847 | C | A | -0.151 | 0.015 | 6.03×10^-11 |
| UC | rs1801274 | G | A | -0.174 | 0.016 | 3.78×10^-17 |
| UC | rs7554511 | A | C | -0.163 | 0.017 | 1.05×10^-11 |
| UC | rs3024505 | A | G | 0.223 | 0.013 | 2.97×10^-17 |
| UC | rs7608910 | G | A | 0.131 | 0.012 | 1.81×10^-12 |
| UC | rs3749171 | A | G | 0.140 | 0.014 | 2.33×10^-10 |
| UC | rs9868809 | A | G | 0.148 | 0.018 | 6.01×10^-09 |
| UC | rs113010081 | G | A | 0.131 | 0.018 | 9.02×10^-10 |
| UC | rs2189234 | A | C | 0.077 | 0.009 | 1.95×10^-10 |
| UC | rs254560 | A | G | 0.077 | 0.012 | 1.6×10^-08 |
| UC | rs56167332 | A | C | 0.140 | 0.011 | 5.3×10^-11 |
| UC | rs6920220 | A | G | 0.148 | 0.013 | 5.22×10^-11 |
| UC | rs7805114 | C | A | -0.117 | 0.015 | 5.86×10^-09 |
| UC | rs1077773 | G | A | -0.073 | 0.011 | 5.96×10^-09 |
| UC | rs4246905 | A | G | -0.117 | 0.016 | 1.97×10^-08 |
| UC | rs10781499 | A | G | 0.131 | 0.012 | 7.69×10^-13 |
| UC | rs4409764 | A | C | 0.157 | 0.011 | 8.39×10^-15 |
| UC | rs2155219 | A | C | 0.122 | 0.012 | 1.48×10^-09 |
| UC | rs483905 | A | G | 0.086 | 0.013 | 1.57×10^-08 |
| UC | rs561722 | A | G | -0.128 | 0.016 | 5.21×10^-09 |
| UC | rs7134472 | A | G | 0.157 | 0.011 | 4.77×10^-18 |
| UC | rs17085007 | G | A | 0.131 | 0.014 | 1.18×10^-08 |
| UC | rs12946510 | A | G | 0.131 | 0.012 | 4.95×10^-10 |
| UC | rs17736589 | G | A | 0.086 | 0.019 | 4.34×10^-08 |
| UC | rs2836878 | A | G | -0.223 | 0.019 | 2.05×10^-20 |
| UC | rs7282490 | G | A | 0.104 | 0.012 | 7.08×10^-11 |

# **Table S1 Definitions of IBD and IBD-related clinical outcomes**

| **Outcomes** | **Diagnosis code/description** |
| --- | --- |
| **Inflammatory Bowel Disease** | ICD-9: 555, 556  ICD-10: K50, K51 |
| **Cardiovascular diseases**  (PMID: 24217719, 16020772, 31738818) | ICD-9: 410-414, 362.3, 430-431, 433-436, 440, 443.9  ICD-10: I20-I25, H34.1, I60-I61, I63-I64, G45, I70, I73.9 |
| **Diabetes Mellitus**  (PMID: 31738818) | ICD-9: 250  ICD-10: E10-E14 |
| **IBD-related surgery**  (PMID: 22070187) | Including: Colorectal/large bowel resection; small bowel and colorectal resection; small bowel resection; surgery for perineal disease. |
| **All-cause mortality** | Extracting from death register in UK Biobank (https://biobank.ndph.ox.ac.uk/showcase/field.cgi?id=40000). |

# **Table S2 Characteristics of participants with IBD by healthy plant-based diet index quintiles in the UK Biobank**

| **Characteristics** | **Overall** | **Quintile 1** | **Quintile 2** | **Quintile 3** | **Quintile 4** | **Quintile 5** |
| --- | --- | --- | --- | --- | --- | --- |
|  | N=2133 | N=437 | N=428 | N=427 | N=459 | N=382 |
| Mean healthy PDI (SD) | 55.7 (6.2) | 47.1 (3.0) | 52.6 (1.0) | 55.9 (0.9) | 59.2 (1.1) | 64.7 (2.9) |
| Age (SD) | 56.8 (7.8) | 55.6 (8.3) | 57.0 (7.9) | 56.6 (7.9) | 57.5 (7.3) | 57.4 (7.5) |
| Female (%) | 1,091 (51.1) | 161 (36.8) | 205 (47.9) | 208 (48.7) | 269 (58.6) | 248 (64.9) |
| White (%) | 2,056 (96.4) | 418 (95.7) | 417 (97.4) | 410 (96.0) | 445 (96.9) | 366 (95.8) |
| TDI (SD) | -1.5 (2.9) | -1.2 (3.0) | -1.8 (2.8) | -1.7 (2.8) | -1.5 (2.9) | -1.6 (2.8) |
| With university/college degree (%) | 787 (37.1) | 129 (29.7) | 157 (36.9) | 170 (39.9) | 183 (40.2) | 148 (39.1) |
| BMI (SD, kg/m^2^) | 26.7 (4.6) | 27.3 (4.8) | 26.9 (4.6) | 26.6 (4.9) | 26.8 (4.4) | 25.9 (4.0) |
| Never smoking (%) | 1,048 (49.2) | 204 (46.7) | 203 (47.4) | 225 (53.1) | 221 (48.1) | 195 (51.3) |
| Exercise time (SD, minutes per day) | 11.4 (8.6) | 10.8 (8.8) | 11.4 (8.1) | 10.9 (8.6) | 11.6 (8.6) | 12.2 (9.1) |
| CCI (SD) | 0.4 (1.0) | 0.4 (1.1) | 0.4 (1.1) | 0.4 (1.1) | 0.3 (1.0) | 0.3 (1.0) |
| CRP (SD, mg/L) | 3.6 (5.9) | 4.1 (6.1) | 4.1 (5.9) | 3.6 (7.2) | 3.3 (5.0) | 2.9 (5.1) |
| INFLA score (SD) | 1.7 (6.4) | 3.1 (6.0) | 2.2 (6.5) | 1.3 (6.4) | 1.5 (6.2) | 0.5 (6.5) |
| Alcohol intake (SD, g/d) | 13.4 (19.1) | 11.3 (18.4) | 14.7 (19.0) | 13.8 (19.4) | 15.1 (19.8) | 12.1 (18.4) |
| Total sugar (SD, g/d) | 11.4 (8.6) | 10.8 (8.8) | 11.4 (8.1) | 10.9 (8.6) | 11.6 (8.6) | 12.2 (9.1) |
| Total energy (SD, kj/d) | 8,834.4 (2,343.3) | 9,958.7 (2,313.5) | 9,248.6 (2,246.4) | 8,438.4 (2,095.5) | 8,395.8 (2,313.1) | 8,053.6 (2,223.3) |

BMI, body mass index; CCI, Charlson comorbidity index; CRP, C-reactive protein; TDI, Townsend deprivation index;

# **Table S3 Associations of** **plant-based diet indexes with clinical outcomes among individuals with Crohn’s disease and ulcerative colitis in the UK Biobank**

| **Clinical outcomes (total population)** | **Plant-based diet indexes** | **Individuals with CD** | | | **Individuals with UC** | | |
| --- | --- | --- | --- | --- | --- | --- | --- |
|  |  | **Cases/**  **Person-years** | **HR (95% CI)** | ***P* value** | **Cases/**  **Person-years** | **HR (95% CI)** | ***P* value** |
| **CVD**  **(CD=431, UC=1009)** | **PDI** |  |  |  |  |  |  |
|  | Quintile 1 | 8/1,039 | Reference |  | 15/2,058 | Reference |  |
|  | Quintile 2 | 7/1,037 | 0.49 (0.17, 1.43) | 0.193 | 25/2,245 | 1.47 (0.77, 2.79) | 0.243 |
|  | Quintile 3 | 6/1,123 | 0.51 (0.15, 1.69) | 0.268 | 32/2,846 | 1.42 (0.76, 2.65) | 0.27 |
|  | Quintile 4 | 4/833 | 0.55 (0.14, 2.19) | 0.397 | 22/2,029 | 1.34 (0.68, 2.62) | 0.395 |
|  | Quintile 5 | 3/828 | 0.21 (0.05, 0.92) | **0.038** | 20/1,785 | 1.32 (0.65, 2.68) | 0.451 |
|  | *P* for trend |  |  | 0.059 |  |  | 0.618 |
|  | Per 10 units | | 0.86 (0.35, 2.09) | 0.735 |  | 1.15 (0.75, 1.75) | 0.516 |
|  | **Healthy PDI** | |  |  |  |  |  |
|  | Quintile 1 | 9/1,024 | Reference |  | 17/2,000 | Reference |  |
|  | Quintile 2 | 9/1,036 | 0.99 (0.38, 2.63) | 0.992 | 23/1,996 | 1.45 (0.77, 2.75) | 0.248 |
|  | Quintile 3 | 2/1,143 | 0.37 (0.07, 1.83) | 0.223 | 25/2,171 | 1.45 (0.76, 2.76) | 0.264 |
|  | Quintile 4 | 5/983 | 0.73 (0.22, 2.47) | 0.618 | 23/2,430 | 1.31 (0.68, 2.53) | 0.421 |
|  | Quintile 5 | 3/674 | 0.56 (0.14, 2.25) | 0.417 | 26/2,367 | 1.48 (0.77, 2.86) | 0.244 |
|  | *P* for trend |  |  | 0.313 |  |  | 0.373 |
|  | Per 10 units | | 0.80 (0.42, 1.52) | 0.491 |  | 1.07 (0.78, 1.46) | 0.688 |
|  | **Unhealthy PDI** | |  |  |  |  |  |
|  | Quintile 1 | 5/775 | Reference |  | 26/2,524 | Reference |  |
|  | Quintile 2 | 3/984 | 0.44 (0.10, 1.95) | 0.279 | 22/2,110 | 1.03 (0.58, 1.82) | 0.931 |
|  | Quintile 3 | 4/1,037 | 0.96 (0.25, 3.68) | 0.955 | 22/2,206 | 0.92 (0.51, 1.63) | 0.765 |
|  | Quintile 4 | 9/1,103 | 1.09 (0.35, 3.42) | 0.880 | 27/2,418 | 1.02 (0.59, 1.77) | 0.934 |
|  | Quintile 5 | 7/960 | 1.80 (0.54, 5.99) | 0.339 | 17/1,706 | 1.03 (0.55, 1.95) | 0.922 |
|  | *P* for trend |  |  | 0.168 |  |  | 0.947 |
|  | Per 10 units | | 1.52 (0.78, 2.99) | 0.222 |  | 0.97 (0.71, 1.33) | 0.859 |
| **Diabetes Mellitus**  **(CD=592, UC=1406)** | **PDI** |  |  |  |  |  |  |
|  | Quintile 1 | 6/1,466 | Reference |  | 13/2,952 | Reference |  |
|  | Quintile 2 | 10/1,402 | 1.33 (0.47, 3.77) | 0.590 | 15/3,156 | 0.90 (0.42, 1.90) | 0.774 |
|  | Quintile 3 | 4/1,504 | 0.33 (0.08, 1.48) | 0.149 | 26/4,072 | 1.32 (0.67, 2.61) | 0.429 |
|  | Quintile 4 | 3/1,261 | 0.48 (0.11, 2.06) | 0.324 | 19/2,955 | 1.16 (0.56, 2.39) | 0.684 |
|  | Quintile 5 | 3/1,034 | 0.44 (0.10, 2.05) | 0.299 | 18/2,514 | 1.31 (0.62, 2.80) | 0.481 |
|  | *P* for trend |  |  | 0.133 |  |  | 0.358 |
|  | Per 10 units | | 0.71 (0.28, 1.79) | 0.471 |  | 1.20 (0.76, 1.91) | 0.437 |
|  | **Healthy PDI** | |  |  |  |  |  |
|  | Quintile 1 | 7/1,440 | Reference |  | 18/3,044 | Reference |  |
|  | Quintile 2 | 7/1,436 | 1.20 (0.41, 3.56) | 0.740 | 21/2,971 | 1.38 (0.72, 2.64) | 0.329 |
|  | Quintile 3 | 2/1,488 | 0.38 (0.07, 1.94) | 0.244 | 24/2,945 | 1.47 (0.77, 2.82) | 0.246 |
|  | Quintile 4 | 4/1,334 | 0.59 (0.16, 2.14) | 0.418 | 14/3,641 | 0.79 (0.38, 1.65) | 0.530 |
|  | Quintile 5 | 6/970 | 1.47 (0.46, 4.74) | 0.517 | 14/3,048 | 1.25 (0.58, 2.68) | 0.569 |
|  | *P* for trend |  |  | 0.872 |  |  | 0.969 |
|  | Per 10 units |  | 1.16 (0.59, 2.28) | 0.658 |  | 1.03 (0.71, 1.50) | 0.881 |
|  | **Unhealthy PDI** | |  |  |  |  |  |
|  | Quintile 1 | 3/1,184 | Reference |  | 16/3,436 | Reference |  |
|  | Quintile 2 | 9/1,325 | 2.49 (0.65, 9.51) | 0.181 | 17/3,203 | 1.24 (0.61, 2.50) | 0.549 |
|  | Quintile 3 | 3/1,397 | 0.89 (0.17, 4.53) | 0.888 | 12/3,228 | 0.82 (0.38, 1.77) | 0.614 |
|  | Quintile 4 | 3/1,339 | 0.82 (0.16, 4.17) | 0.812 | 30/3,424 | 1.86 (0.99, 3.48) | 0.054 |
|  | Quintile 5 | 8/1,423 | 1.32 (0.31, 5.67) | 0.709 | 16/2,357 | 1.51 (0.74, 3.09) | 0.256 |
|  | *P* for trend |  |  | 0.728 |  |  | 0.103 |
|  | Per 10 units | | 0.88 (0.43, 1.76) | 0.709 |  | 1.26 (0.89, 1.80) | 0.193 |
| **IBD-related surgery**  **(CD=631, UC=1478)** | **PDI** |  |  |  |  |  |  |
|  | Quintile 1 | 13/1,303 | Reference |  | 18/2,619 | Reference |  |
|  | Quintile 2 | 12/1,220 | 0.89 (0.40, 1.95) | 0.762 | 22/2,773 | 1.10 (0.58, 2.06) | 0.773 |
|  | Quintile 3 | 19/1,248 | 1.24 (0.61, 2.55) | 0.553 | 30/3,589 | 1.19 (0.66, 2.17) | 0.563 |
|  | Quintile 4 | 18/1,033 | 1.27 (0.60, 2.69) | 0.527 | 15/2,688 | 0.75 (0.37, 1.51) | 0.424 |
|  | Quintile 5 | 11/908 | 0.75 (0.32, 1.78) | 0.517 | 18/2,263 | 1.01 (0.50, 2.05) | 0.978 |
|  | *P* for trend |  |  | 0.864 |  |  | 0.658 |
|  | Per 10 units | | 0.85 (0.51, 1.44) | 0.555 |  | 0.89 (0.57, 1.39) | 0.612 |
|  | **Healthy PDI** | |  |  |  |  |  |
|  | Quintile 1 | 23/1,277 | Reference |  | 33/2,589 | Reference |  |
|  | Quintile 2 | 22/1,242 | 1.10 (0.60, 2.01) | 0.768 | 22/2,623 | 0.69 (0.40, 1.20) | 0.191 |
|  | Quintile 3 | 17/1,202 | 0.93 (0.48, 1.79) | 0.824 | 19/2,704 | 0.60 (0.33, 1.09) | 0.093 |
|  | Quintile 4 | 5/1,140 | 0.32 (0.12, 0.86) | **0.024** | 12/3,266 | 0.32 (0.16, 0.63) | **0.001** |
|  | Quintile 5 | 6/851 | 0.41 (0.16, 1.04) | 0.062 | 17/2,750 | 0.53 (0.28, 1.01) | 0.054 |
|  | *P* for trend |  |  | **0.010** |  |  | **0.007** |
|  | Per 10 units | | 0.69 (0.47, 1.02) | 0.063 |  | 0.62 (0.45, 0.88) | **0.006** |
|  | **Unhealthy PDI** | |  |  |  |  |  |
|  | Quintile 1 | 6/1,050 | Reference |  | 22/3,052 | Reference |  |
|  | Quintile 2 | 13/1,171 | 1.98 (0.75, 5.27) | 0.169 | 20/2,897 | 0.98 (0.53, 1.80) | 0.945 |
|  | Quintile 3 | 17/1,144 | 2.74 (1.07, 7.01) | **0.035** | 13/2,848 | 0.65 (0.33, 1.31) | 0.229 |
|  | Quintile 4 | 18/1,173 | 2.76 (1.08, 7.03) | **0.033** | 22/3,106 | 1.00 (0.55, 1.82) | 0.998 |
|  | Quintile 5 | 19/1,173 | 2.79 (1.08, 7.17) | **0.034** | 26/2,029 | 1.82 (1.01, 3.26) | **0.046** |
|  | *P* for trend |  |  | **0.029** |  |  | 0.082 |
|  | Per 10 units | | 1.46 (0.99, 2.14) | 0.057 |  | 1.39 (0.99, 1.93) | 0.056 |
| **All-cause mortality**  **(CD=631, UC=1478)** | **PDI** |  |  |  |  |  |  |
|  | Quintile 1 | 11/1,372 | Reference |  | 19/2,718 | Reference |  |
|  | Quintile 2 | 3/1,279 | 0.31 (0.09, 1.14) | 0.079 | 27/2,892 | 1.38 (0.76, 2.50) | 0.288 |
|  | Quintile 3 | 7/1,349 | 0.55 (0.20, 1.47) | 0.234 | 27/3,742 | 1.08 (0.59, 1.97) | 0.795 |
|  | Quintile 4 | 7/1,131 | 0.67 (0.25, 1.80) | 0.427 | 15/2,785 | 0.77 (0.39, 1.54) | 0.462 |
|  | Quintile 5 | 11/966 | 1.26 (0.49, 3.28) | 0.629 | 18/2,346 | 1.14 (0.57, 2.28) | 0.705 |
|  | *P* for trend |  |  | 0.527 |  |  | 0.646 |
|  | Per 10 units | | 1.21 (0.58, 2.51) | 0.617 |  | 0.92 (0.59, 1.43) | 0.706 |
|  | **Healthy PDI** | |  |  |  |  |  |
|  | Quintile 1 | 10/1,407 | Reference |  | 19/2,783 | Reference |  |
|  | Quintile 2 | 11/1,349 | 1.16 (0.47, 2.83) | 0.752 | 27/2,736 | 1.37 (0.75, 2.49) | 0.304 |
|  | Quintile 3 | 10/1,299 | 1.12 (0.43, 2.90) | 0.818 | 21/2,813 | 1.01 (0.53, 1.93) | 0.971 |
|  | Quintile 4 | 2/1,163 | 0.23 (0.05, 1.06) | 0.059 | 18/3,321 | 0.75 (0.38, 1.48) | 0.414 |
|  | Quintile 5 | 6/879 | 0.92 (0.31, 2.69) | 0.872 | 21/2,830 | 1.07 (0.54, 2.09) | 0.854 |
|  | *P* for trend |  |  | 0.325 |  |  | 0.584 |
|  | Per 10 units | | 0.81 (0.47, 1.40) | 0.443 |  | 0.90 (0.64, 1.26) | 0.531 |
|  | **Unhealthy PDI** | |  |  |  |  |  |
|  | Quintile 1 | 7/1,075 | Reference |  | 23/3,160 | Reference |  |
|  | Quintile 2 | 10/1,235 | 1.24 (0.46, 3.36) | 0.676 | 21/3,011 | 0.93 (0.52, 1.69) | 0.822 |
|  | Quintile 3 | 6/1,241 | 0.81 (0.27, 2.46) | 0.710 | 17/2,915 | 0.73 (0.38, 1.37) | 0.323 |
|  | Quintile 4 | 10/1,261 | 1.29 (0.48, 3.49) | 0.617 | 24/3,237 | 1.05 (0.59, 1.87) | 0.867 |
|  | Quintile 5 | 6/1,285 | 0.73 (0.23, 2.31) | 0.595 | 21/2,160 | 1.62 (0.88, 2.98) | 0.119 |
|  | *P* for trend |  |  | 0.644 |  |  | 0.108 |
|  | Per 10 units | | 0.97 (0.56, 1.70) | 0.927 |  | 1.32 (0.94, 1.86) | 0.110 |

CVD, cardiovascular disease, PDI, plant-based diet index; HR, hazard ratio; CI, confidence interval.

^a^ based on the fully adjusted model

# **Table S4 Associations of** **healthy omnivorous diet with incident inflammatory bowel disease in the UK Biobank ^a^**

| **Healthy omnivorous diet score in quintiles** | **Cases/person-years** | **HR (95% CI)^a^** | ***P* value** | **HR (95% CI) ^b^** | ***P* value** |
| --- | --- | --- | --- | --- | --- |
| Q1 (43.3, <=46.0) | 189/426,540 | Reference |  |  |  |
| Q2 (48.5, >46.0-<=50.0) | 214/444,805 | 1.01 (0.84, 1.21) | 0.946 | 1.00 (0.83, 1.20) | 0.998 |
| Q3 (52.0, >50.0-<=53.0) | 206/490,418 | 0.83 (0.68, 1.02) | 0.074 | 0.83 (0.67, 1.03) | 0.089 |
| Q4 (55.0, >53.0-<=57.0) | 153/421,458 | **0.81 (0.66, 1.00)** | **0.047** | 0.82 (0.66, 1.02) | 0.068 |
| Q5 (60.0, >57.0) | 163/358,478 | **0.71 (0.56, 0.89)** | **0.003** | **0.71 (0.55, 0.91)** | **0.007** |
| *P* for trend | |  | **0.0005** |  | **0.002** |
| Per 10 units | | **0.84 (0.75, 0.93)** | **0.001** | **0.84 (0.75, 0.95)** | **0.006** |

PDI, plant-based diet index; HR, hazard ratio; CI, confidence interval.

^a^ based on the fully adjusted model

^b^ additionally adjusted for dietary fibre intake based on the fully adjusted model


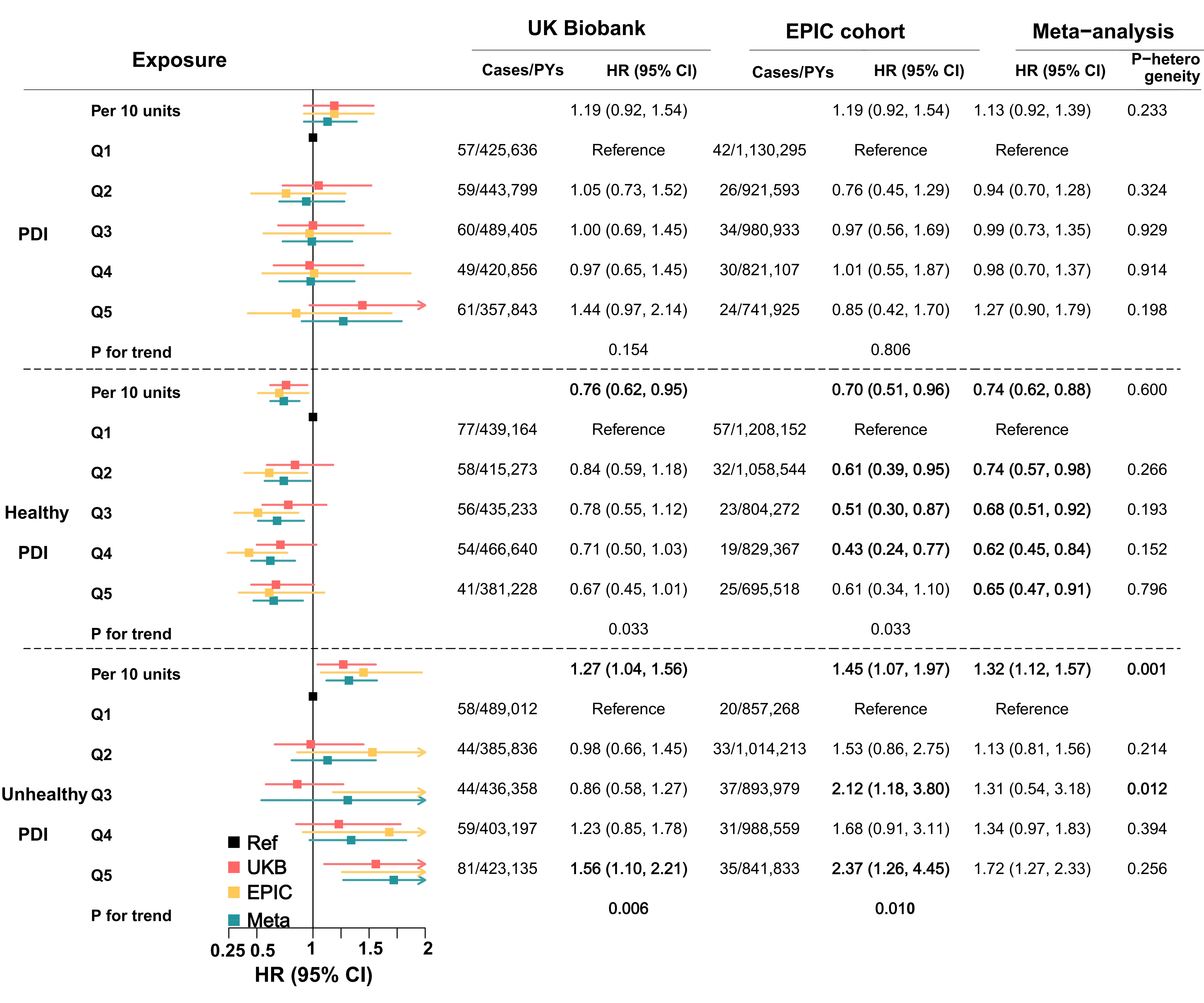


# **Figure S1 Associations between the plant-based diet indexes and incident Crohn’s disease in UK Biobank (n=187,888), EPIC cohort (n=341,539), and the meta-analysis of the results from the two cohorts (n=529,427).** HRs were adjusted for age, sex, Townsend deprivation index, education, ethnicity, body mass index, smoking status, alcohol consumption, exercise time, total energy intake and total sugar intake in UK Biobank. HRs were adjusted for age, sex, center, education level, BMI, smoking status, alcohol consumption, total sugar, total energy, and physical activity measured in the EPIC study. The 20%, 40%, 60% and 80% percentile of PDIs in the UK Biobank and EPIC study were used as cut-off values as follow: PDI: (UKB) 46.8, 49.5, 52.0, 55.0; (EPIC) 44.0, 48.0, 52.0, 56.0; healthy PDI: (UKB) 52.0, 55.7, 58.5, 62.0; (EPIC) 46.0, 50.0, 53.0, 57.0; unhealthy PDI: (UKB) 52.0, 55.0, 58.0, 61.5; (EPIC) 48.0, 52.0, 55.0, 59.0. Numbers in bold indicates significant associations. P for heterogeneity were tested using Cochran Q test. PDI, plant-based diet index; HR, hazard ratio; CI, confidence interval; EPIC, European Prospective Investigation into Cancer, and Nutrition; PY, person-years


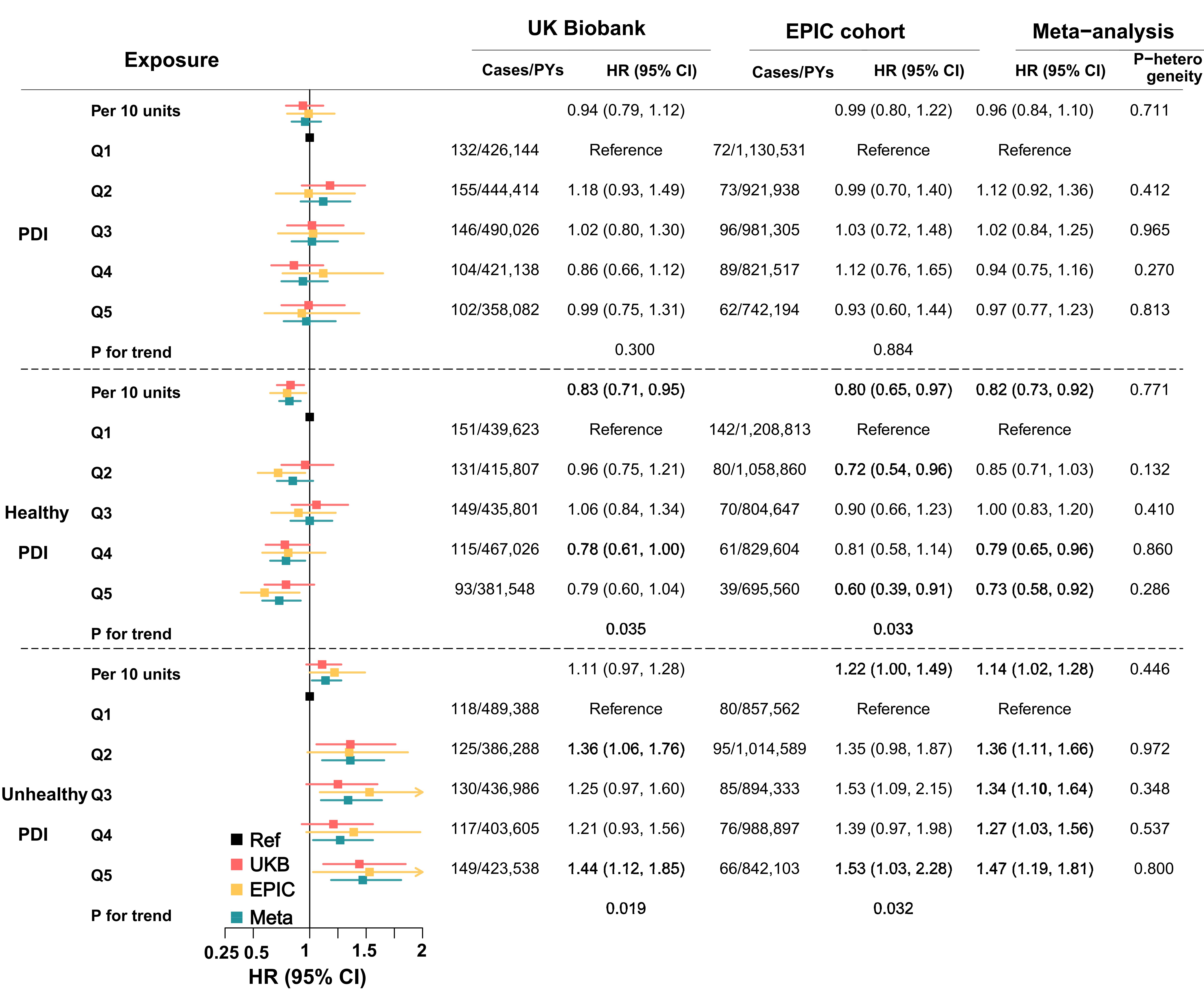


# **Figure S2. Associations between the plant-based diet indexes and incident ulcerative colitis in UK Biobank (n=187,888), EPIC cohort (n=341,539), and the meta-analysis of the results from the two cohorts (n=529,427).** HRs were adjusted for age, sex, Townsend deprivation index, education, ethnicity, body mass index, smoking status, alcohol consumption, exercise time, total energy intake and total sugar intake in UK Biobank. HRs were adjusted for age, sex, center, education level, BMI, smoking status, alcohol consumption, total sugar, total energy, and physical activity measured in the EPIC study. The 20%, 40%, 60% and 80% percentile of PDIs in the UK Biobank and EPIC study were used as cut-off values as follow: PDI: (UKB) 46.8, 49.5, 52.0, 55.0; (EPIC) 44.0, 48.0, 52.0, 56.0; healthy PDI: (UKB) 52.0, 55.7, 58.5, 62.0; (EPIC) 46.0, 50.0, 53.0, 57.0; unhealthy PDI: (UKB) 52.0, 55.0, 58.0, 61.5; (EPIC) 48.0, 52.0, 55.0, 59.0. Numbers in bold indicates significant associations. P for heterogeneity were tested using Cochran Q test. PDI, plant-based diet index; HR, hazard ratio; CI, confidence interval; EPIC, European Prospective Investigation into Cancer, and Nutrition; PY, person-years


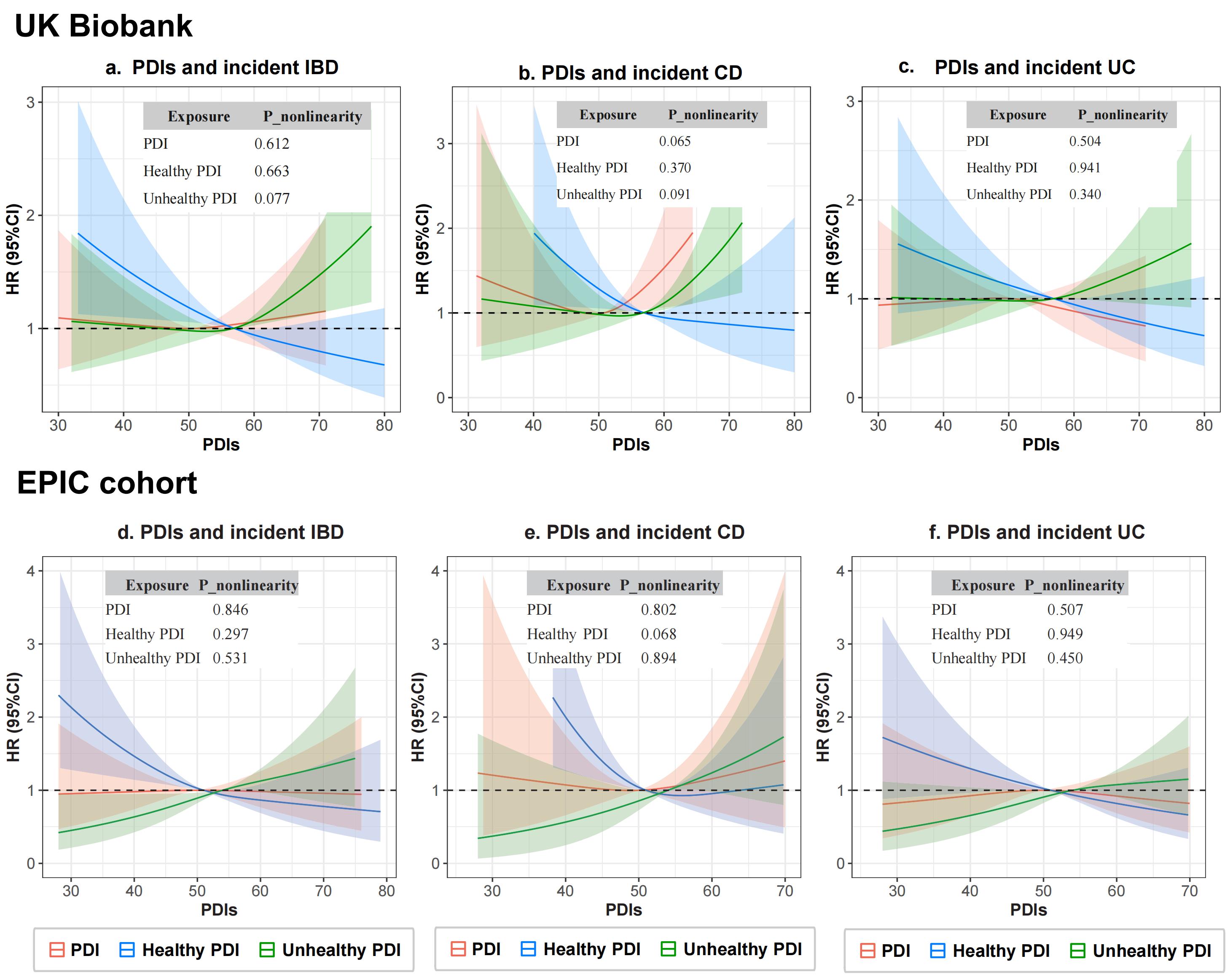


# **Figure S3 Non-linear relationship of the plant-based diet indexes with risk of incident inflammatory bowel disease in the UK Biobank (a-c) and EPIC cohort (d-f).** HRs were adjusted for age, sex, Townsend deprivation index, education, ethnicity, body mass index, smoking status, alcohol consumption, exercise time, total energy intake and total sugar intake in UK Biobank. HRs were adjusted for age, sex, center, education level, BMI, smoking status, alcohol consumption, total sugar, total energy, and physical activity measured in the EPIC study. PDI, plant-based diet index; HR, hazard ratio; CI, confidence interval; EPIC, European Prospective Investigation into Cancer, and Nutrition.


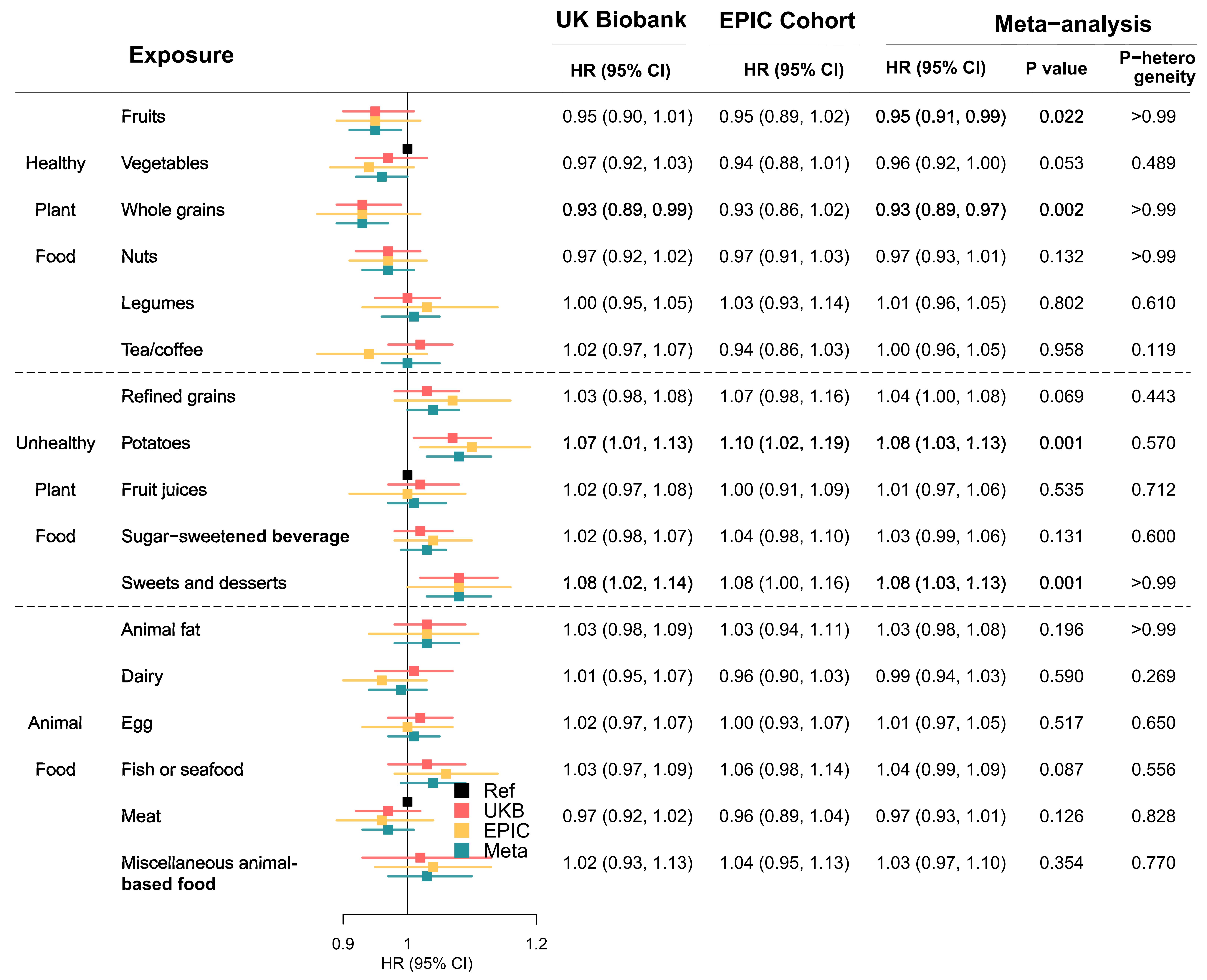


# **Figure S4. Associations between the individual food categories and incident inflammatory bowel disease in UK Biobank (n=187,888), EPIC cohort (n=341,539), and the meta-analysis of the results from the two cohorts (n=529,427).** HRs were adjusted for age, sex, Townsend deprivation index, education, ethnicity, body mass index, smoking status, alcohol consumption, exercise time, total energy intake and total sugar intake in UK Biobank. HRs were adjusted for age, sex, center, education level, BMI, smoking status, alcohol consumption, total sugar, total energy, and physical activity measured in the EPIC study. The Exposure was the subscore of each food group used to calculate the plant-based diet indexes. For each food group subscore, we assigned a score from 1 to 5 based on individual intake quintiles. As mentioned earlier, the same food group was weighted (positively or negatively) differently in different plant-based diet index algorithms. In this analysis, to improve readability, higher subscores imply greater intake of the corresponding food group. Consumption of vegetarian protein substitutes was not taken into account due to the small number of consumers. Numbers in bold indicates significant associations. P for heterogeneity were tested using Cochran Q test. PDI, plant-based diet index; HR, hazard ratio; CI, confidence interval; EPIC, European Prospective Investigation into Cancer, and Nutrition; PY, person-years


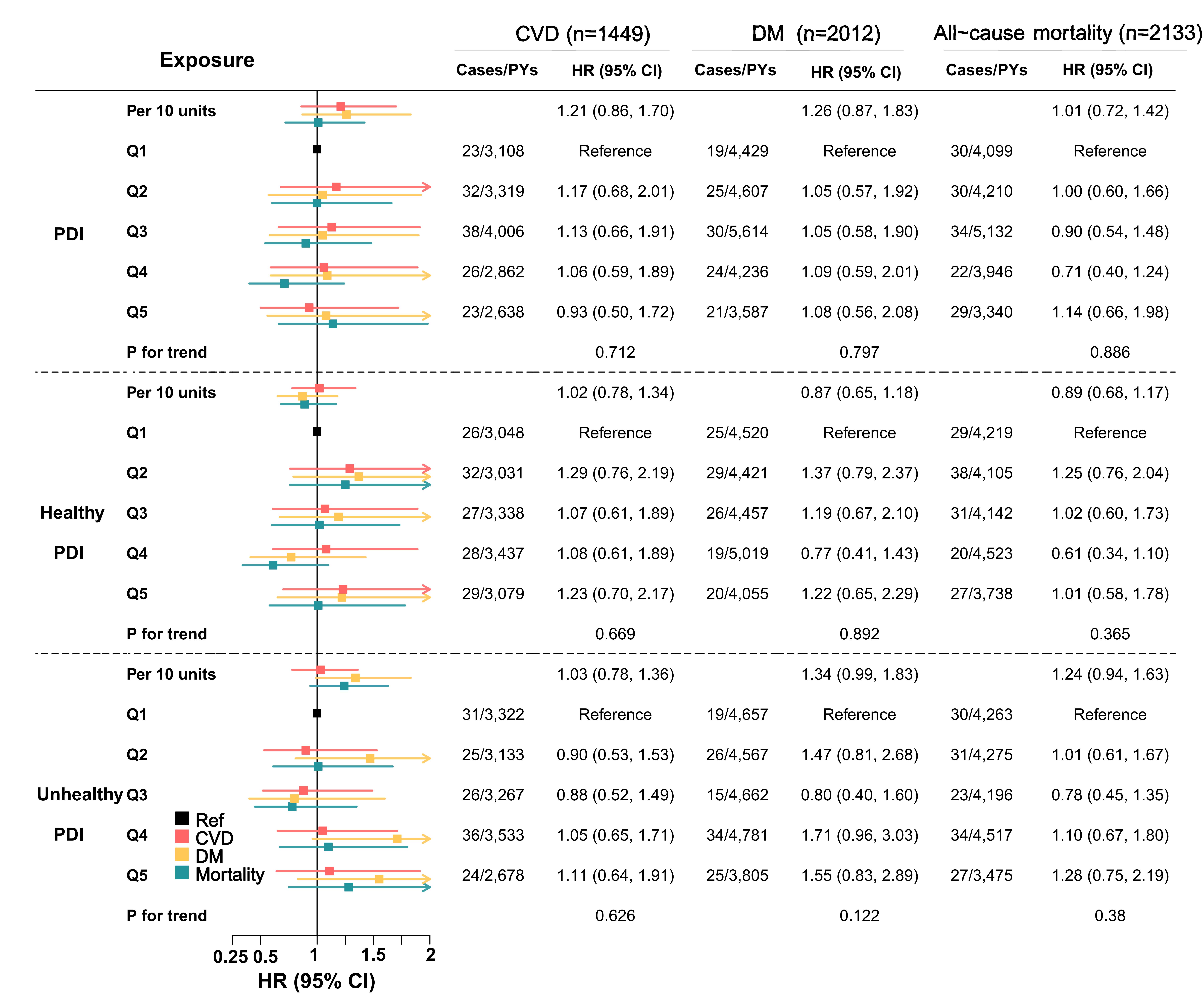


# **Figure S5 Associations of plant-based diet indexes with clinical outcomes (except for IBD-related surgery) among individuals with IBD in the UK Biobank.** Individuals who had an outcome event before baseline were excluded. Model was adjusted for age, sex, Townsend deprivation index, education, ethnicity, body mass index (BMI), smoking status, alcohol consumption, exercise time, total energy intake and total sugar intake. CI, confidence interval; CVD, cardiovascular disease; DM, diabetes mellitus; HR, hazard ratio; PDI, plant-based diet index.


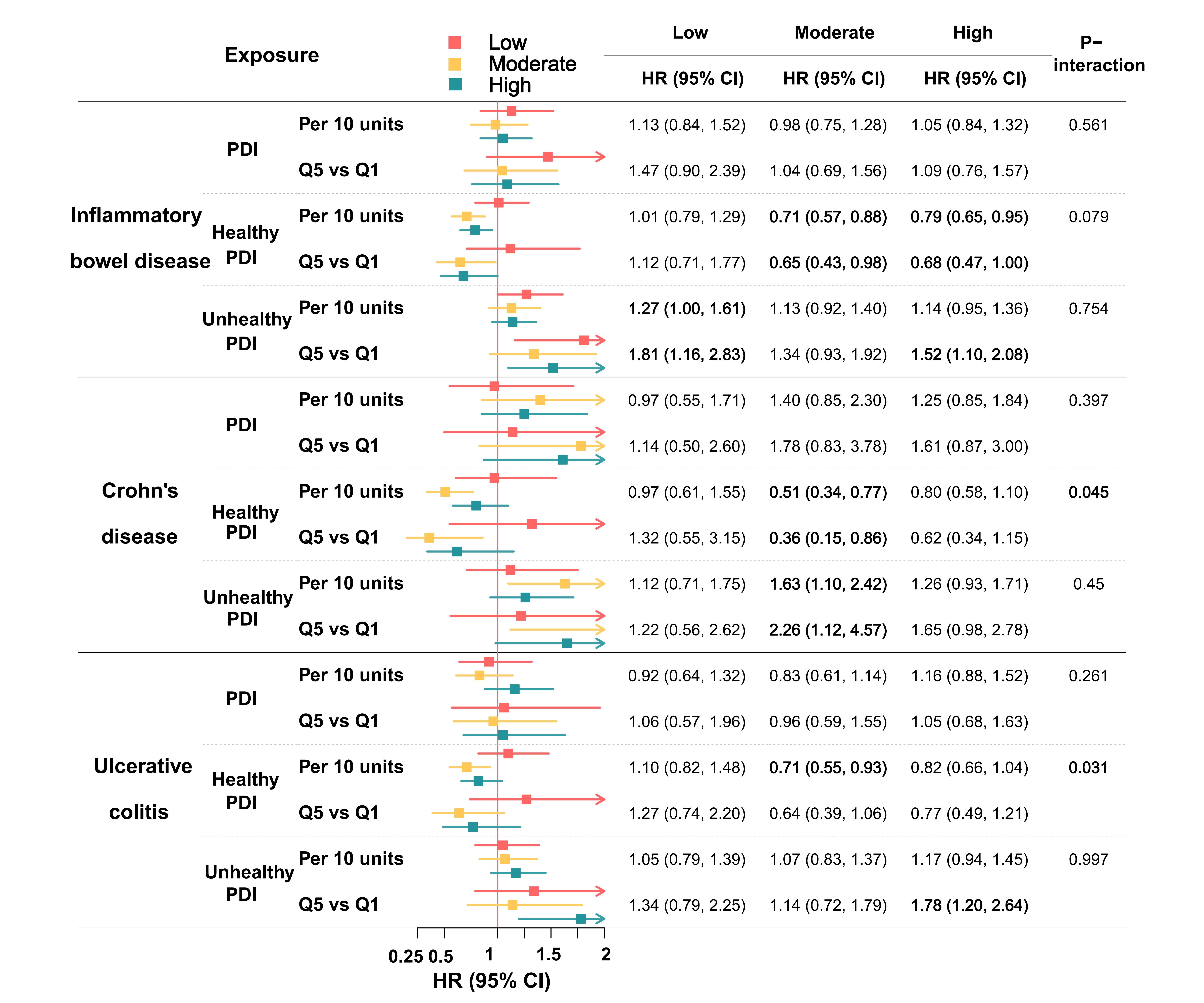


# **Figure S6 Associations of plant-based diet indexes with incident inflammatory bowel disease stratified by genetic risk categories (by tertiles of Polygenic Risk Score) in the UK Biobank.** All models adjusted for age, sex, Townsend deprivation index, education, body mass index (BMI), smoking status, alcohol consumption, exercise time, total energy intake total sugar intake, and the first 5 principal components of ancestry. PDI, plant-based diet index; HR, hazard ratio; CI, confidence interval;


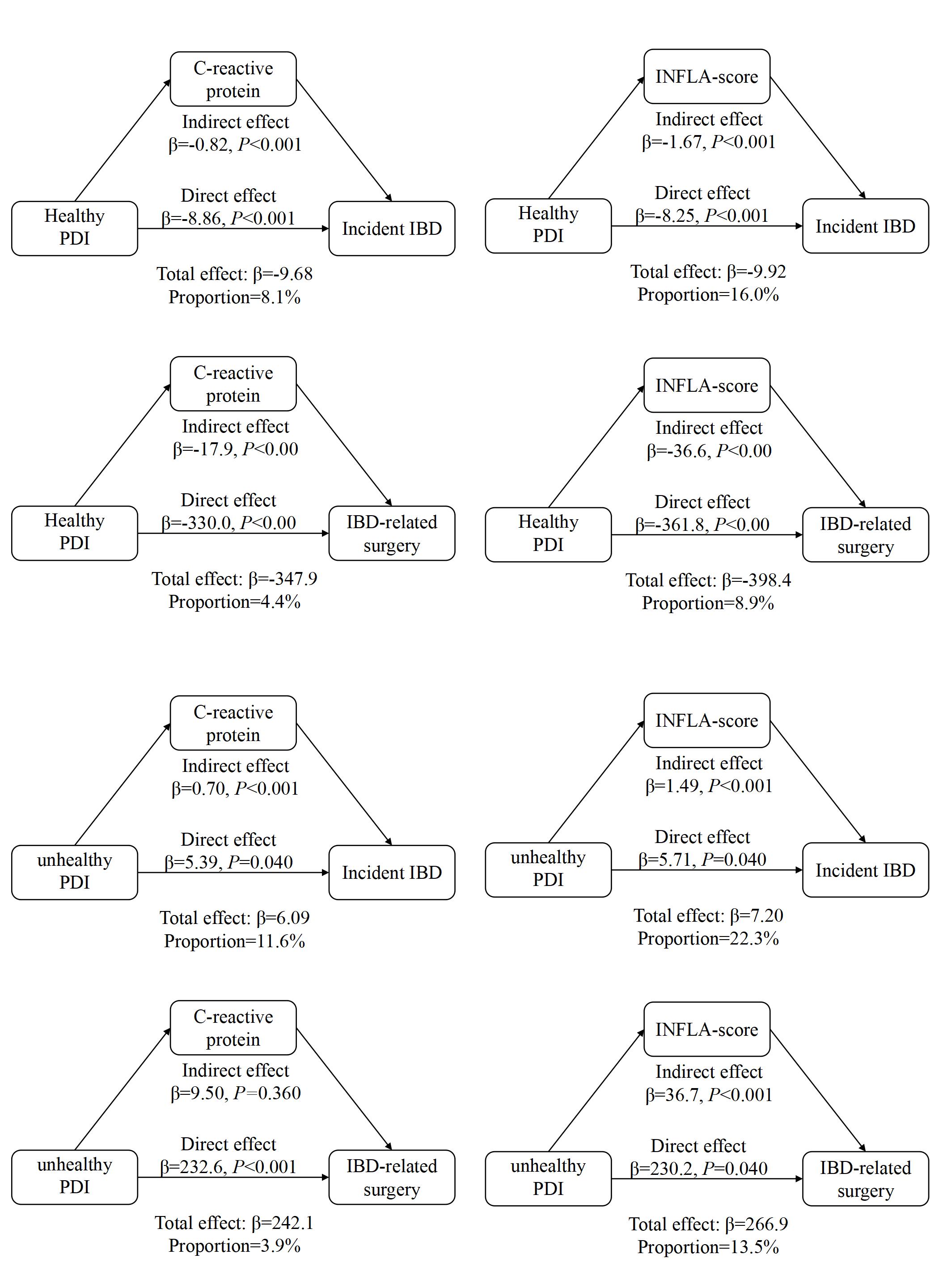


# **Figure S7 mediation analysis for associations between healthy and unhealthy PDI and incident IBD and IBD-related surgery.** The magnitude of the coefficients of the direct and indirect effects is 10^-4^**.** Based on the fully adjusted model; We restricted the analysis to individuals with available CRP levels or INFLA-score. IBD, inflammatory bowel disease, PDI, plant-based diet index; HR, hazard ratio; CI, confidence interval; CRP, C-reactive protein;





# **Figure S8 Associations of plant-based diet indexes with incident IBD (among individuals free of IBD at bassline) and clinical outcomes (among individuals with IBD) in the UK Biobank participants with at least two dietary recalls.** All Models were adjusted for age, sex, Townsend deprivation index, education, ethnicity, body mass index (BMI), smoking status, alcohol consumption, exercise time, total energy intake and total sugar intake. CI, confidence interval; CVD, cardiovascular disease; DM, diabetes mellitus; HR, hazard ratio; PDI, plant-based diet index.


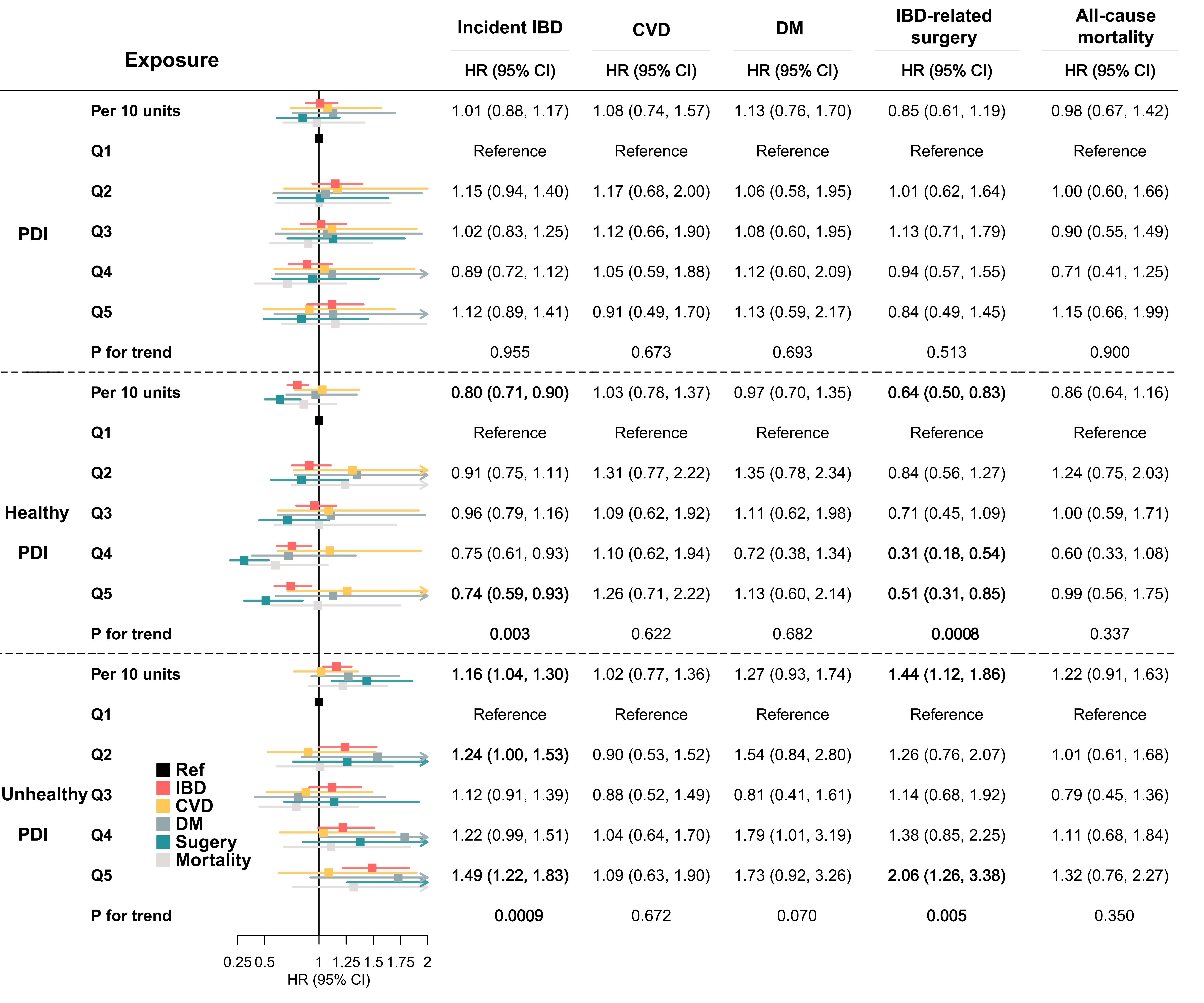


# **Figure S9 Associations of plant-based diet indexes with incident IBD (among individuals free of IBD at bassline) and clinical outcomes (among individuals with IBD) in the UK Biobank participants further adjusted for intake of ultra-processed food.** Based on the fully adjusted model further adjusted for ultra-processed food. CI, confidence interval; CVD, cardiovascular disease; DM, diabetes mellitus; HR, hazard ratio; PDI, plant-based diet index.


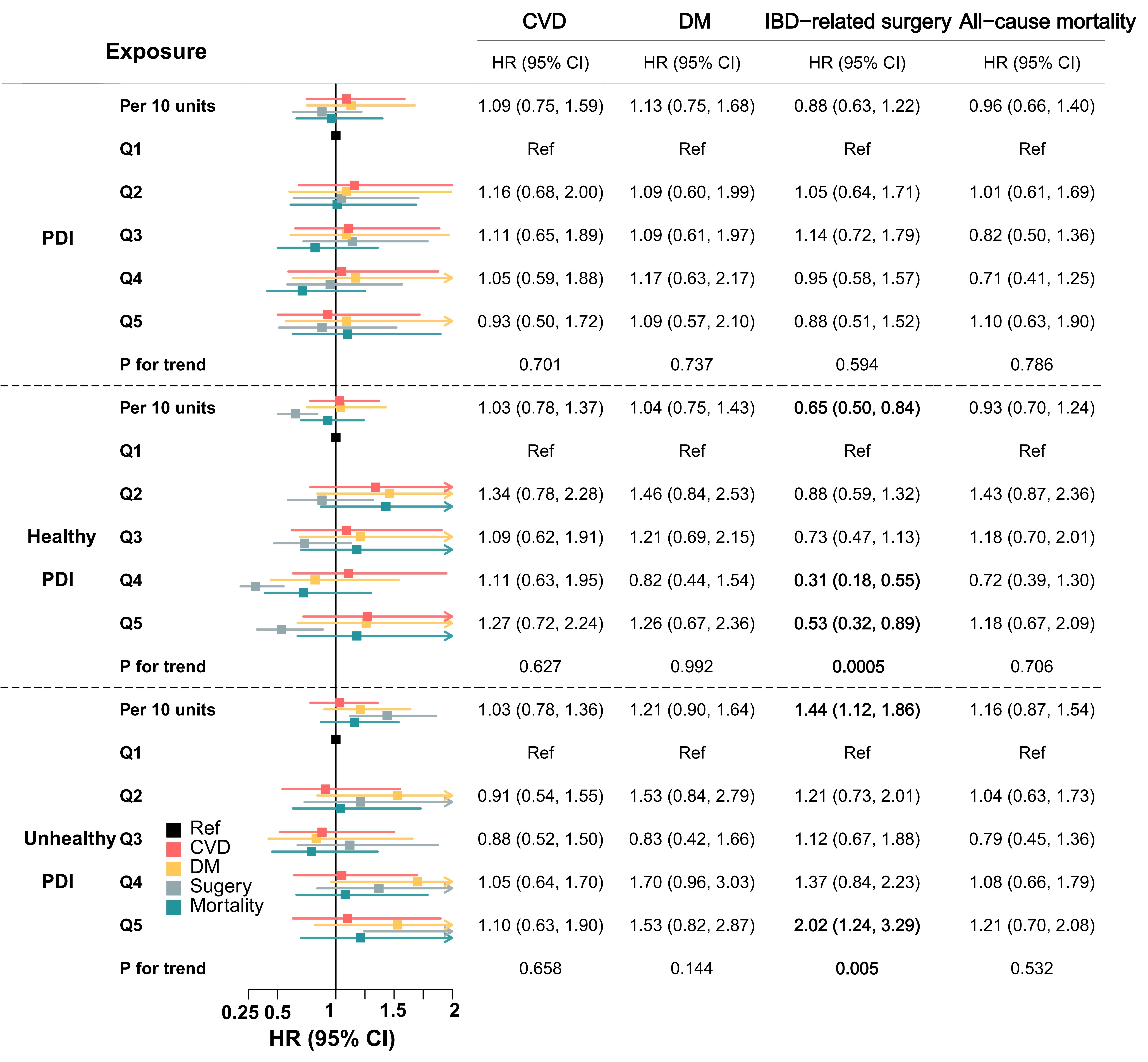


# **Figure S10 Associations of plant-based diet indexes with clinical outcomes (among individuals with IBD) in the UK Biobank participants further adjusted for IBD-related medication** CI, confidence interval; CVD, cardiovascular disease; DM, diabetes mellitus; HR, hazard ratio; PDI, plant-based diet index.


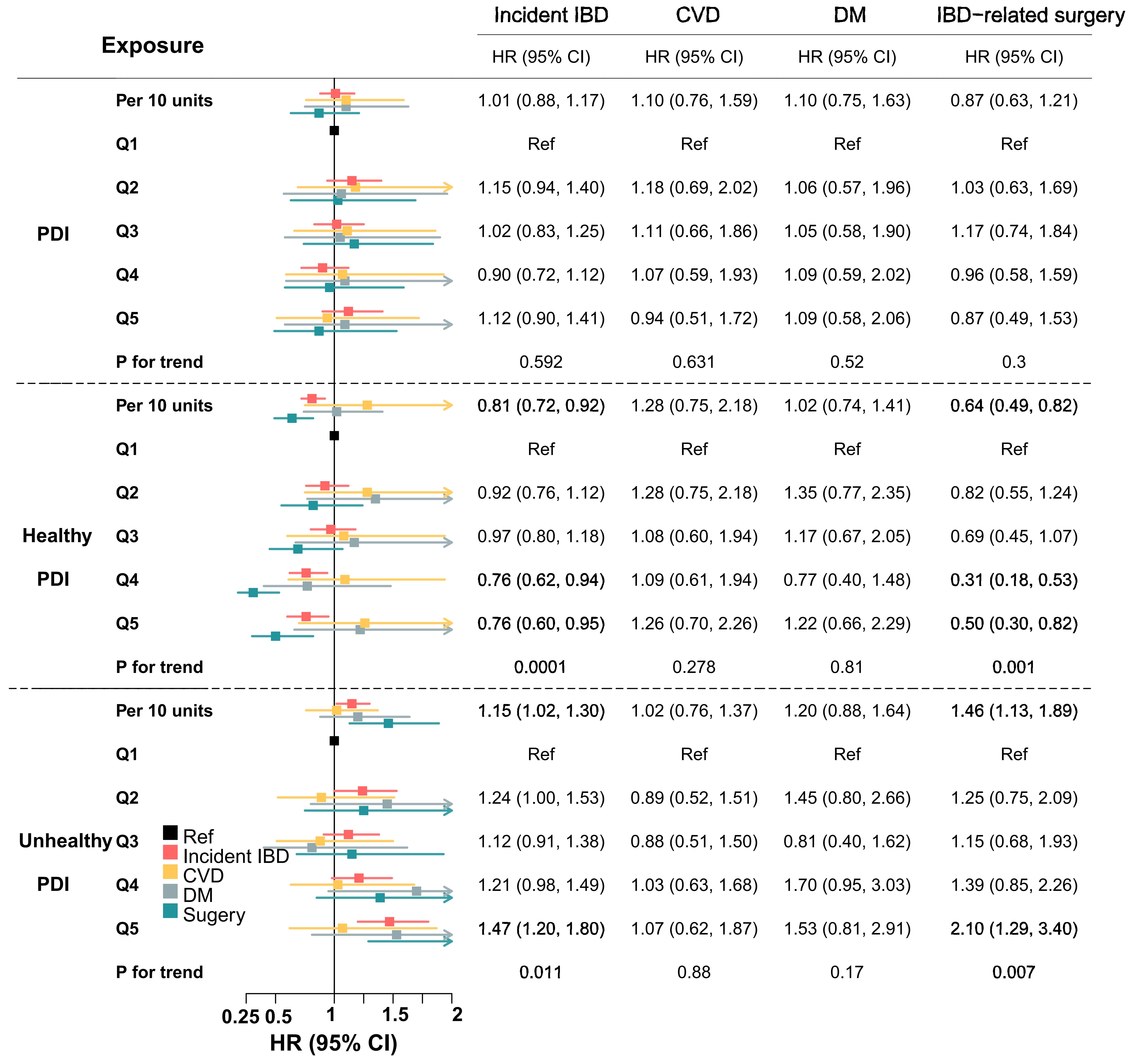


# **Figure S11 Associations of plant-based diet indexes with incident IBD (among individuals free of IBD at bassline) and clinical outcomes (among individuals with IBD) in the UK Biobank participants treating death as competing risk events.** Based on the fully adjusted model further adjusted for ultra-processed food. CI, confidence interval; CVD, cardiovascular disease; DM, diabetes mellitus; HR, hazard ratio; PDI, plant-based diet index.

# **Investigator list of EPIC**

| **First and middle name** | **Surname** | **Affiliation** |
| --- | --- | --- |
| Pilar | Amiano | Public Health Department of the Basque Government, Spain |
| Marie-Christine | Boutron-Ruault | INSERM U1018, Université Paris-Saclay, Villejuif, France |
| Marcela | Guevara | Spanish Consortium for Research on Epidemiology and Public Health (CIBERESP), Instituto de Salud Carlos III, Madrid, Spain |
| Marc J | Gunter | Section of Nutrition and Metabolism, International Agency for Research on Cancer, Lyon, France |
| Mazda | Jenab | International Agency for Research on Cancer, Lyon, France |
| Rudolf | Kaaks | Division of Cancer Epidemiology, German Cancer Research Center (DKFZ), Heidelberg, Germany |
| Tim J | Key | Cancer Epidemiology Unit, Nuffield Department of Clinical Medicine, University of Oxford, Oxford, UK |
| María Dolores Chirlaque | López | Department of Epidemiology and Public Health, University of Murcia, Murcia, Spain |
| Giovanna | Masala | Cancer Risk Factors and Life-Style Epidemiology Unit, Institute for Cancer Research, Prevention and Clinical Network - ISPRO, Florence, Italy |
| Bas | Oldenburg | Julius Center for Health Sciences and Primary Care, University Medical Center Utrecht, Utrecht, The Netherlands |
| Anja | Olsen | Department of Public Health, Faculty of Health and Medical Sciences, University of Copenhagen, Copenhagen, Denmark |
| Elio | Riboli | Department of Epidemiology and Biostatistics, School of Public Health, Imperial College London, London, UK |
| Carlotta | Sacerdote | Unit of Cancer Epidemiology, Città della Salute e della Scienza University, Hospital and Center for Cancer Prevention (CPO), Turin, Italy |
| Matthias | Schulze | Department of Epidemiology, German Institute of Human Nutrition Potsdam-Rehbrücke, Nuthetal, Germany |
| Gianluca | Severi | Centre for Epidemiology and Population Health, INSERM U1018, Université Paris-Saclay, Villejuif, France |
| Anne | Tjønneland | Department of Community Medicine, Faculty of Health Sciences, University of Tromsø, The Arctic University of Norway, Tromsø, Norway |
| Ruth C | Travis | MRC Epidemiology Unit, Institute of Metabolic Science, University of Cambridge, Cambridge, UK |
| Rosario | Tumino | Cancer Registry and Histopathology Department, Civic M.P. Arezzo Hospital, Ragusa, Italy |
| Roel | Vermeulen | Department of Epidemiology, Julius Center for Health Sciences and Primary Care, University Medical Center Utrecht, Utrecht, The Netherlands |
| W. M. Monique Verschuren | W. M. Monique Verschuren | Department of Epidemiology, Julius Center for Health Sciences and Primary Care, University Medical Center Utrecht, Utrecht, The Netherlands |
